# Dissecting genetic variance structure and evaluating genomic prediction models for single-cross hybrids derived from Stiff Stalk and Non-Stiff Stalk maize heterotic groups

**DOI:** 10.1093/g3journal/jkag163

**Published:** 2026-07-03

**Authors:** Jenifer Camila Godoy dos Santos, Jode Edwards, Elizabeth Lee, Mark A Mikel, Samuel B Fernandes, Candice N Hirsch, Sydney P Berry, Alexander E Lipka, Martin O Bohn

**Affiliations:** Department of Crop Sciences, University of Illinois Urbana-Champaign, Urbana, IL 61801, United States; Corn Insects and Crop Genetics Research Unit, United States Department of Agriculture, Agricultural Research Service, Ames, IA 50011-1051, United States; Department of Plant Agriculture, University of Guelph, Guelph, ON, Canada N1G 2W1; Department of Crop Sciences, University of Illinois Urbana-Champaign, Urbana, IL 61801, United States; Crop, Soil, and Environmental Sciences, University of Arkansas, Fayetteville, AR 72701, United States; Department of Agronomy and Plant Genetics, University of Minnesota, Saint Paul, MN 55108, United States; Department of Agronomy and Plant Genetics, University of Minnesota, Saint Paul, MN 55108, United States; Department of Crop Sciences, University of Illinois Urbana-Champaign, Urbana, IL 61801, United States; Department of Crop Sciences, University of Illinois Urbana-Champaign, Urbana, IL 61801, United States

**Keywords:** general combining ability, specific combining ability, heterosis, genomic selection

## Abstract

The early 20th-century discovery of heterosis and the establishment of heterotic groups transformed maize (*Zea mays* L.) into a keystone of global agriculture. However, maize breeding faces two significant challenges: the gradual decline of general combining ability (GCA) variance within heterotic groups and the impracticality of testing all possible single crosses in the early stages of a breeding program. Here, we developed genomic best linear unbiased prediction (GBLUP)-based multikernel models, using additive and two alternative nonadditive genomic relationship matrices, to estimate the variance components associated with the general combining ability of Stiff Stalk (SS) and Non-Stiff Stalk (NSS) heterotic groups and the specific combining ability arising from their crosses. We further applied these models to predict the performance of untested single-cross combinations under varying levels of parental information. We showed that the SS and NSS groups retained significant GCA variance across traits in both early- and late-maturity groups. The SS group, in contrast, exhibited no detectable GCA variance in grain yield for the intermediate-flowering subset of hybrids, highlighting a limitation for future genetic improvement. Furthermore, our results showed that GBLUP-based multikernel models effectively identified superior hybrids when parental information was available. In the absence of this information, however, these models underperformed compared to covariance-based approaches. Both nonadditive matrices yielded similar results, indicating that they capture comparable genetic relationship patterns despite their distinct formulations. Overall, this study sheds light on the future use of US maize commercial germplasm and demonstrates how GBLUP-based multikernel models can improve the efficiency of hybrid breeding programs.

## Introduction

Modern maize breeding has its roots in the early 20th century, when the discovery of heterosis revolutionized hybrid development (East 1908; Shull 1908). Since then, the establishment of heterotic groups has been central to maximizing heterosis. A heterotic group is a collection of inbred lines that are relatively similar to each other but deliver superior hybrid performance when crossed with inbred lines from a contrasting group (Melchinger and Gumber 1998). In US commercial germplasm, one of the most prominent heterotic patterns is the division between the Stiff Stalk (SS) and Non-Stiff Stalk (NSS) groups. The SS group traces its ancestry to the Iowa Stiff Stalk Synthetic population and includes inbred lines such as B37 and B73 (Troyer 1999). The NSS group, further subdivided into Iodent and non-Iodent, consists of genetically diverse sources and encompasses important inbred lines, such as Mo17 and PH207 (Mikel and Dudley 2006).

At its core, maize breeding is a long-term reciprocal recurrent selection program, where two heterotic groups and their intercrosses are improved simultaneously. In practice, this process generally involves crossing inbred lines from one group with testers from the opposite group to estimate the general combining ability (GCA) of the inbred lines and the specific combining ability (SCA) of the resulting hybrids (Sprague and Tatum 1942). The GCA can reflect additive effects and indicates how consistently a parental line contributes to superior hybrid performance across various crosses. By estimating GCA, breeders can identify the most promising inbred lines to be selected and recombined, thereby promoting the accumulation of favorable alleles and progressively enhancing the genetic potential of each heterotic group. At the same time, the preferential selection and eventual fixation of different alleles across groups increase their genetic distance, reinforcing the heterotic pattern. In contrast, SCA captures the hybrid’s heterotic response due to nonadditive effects that frequently involve dominance and/or epistasis (Garcia et al. 2025). A pronounced SCA indicates that the hybrid confers an advantageous performance that cannot be explained solely by the GCA of its parents. Thus, hybrid performance is determined by the combined contribution of parental GCA and their SCA, and the joint evaluation of both is crucial for identifying superior hybrids and advancing them toward commercial release (Comstock et al. 1949; Bernardo 2002).

A significant challenge in maize breeding is the gradual loss of additive genetic variance within heterotic groups, which occurs due to the high intensity of selection implemented over multiple breeding cycles (Fu 2015; Vieira et al. 2025). This issue is particularly pronounced in commercial heterotic groups with a narrow genetic base, such as the SS group, which was established from a limited number of founder inbred lines (Troyer 1999). Although these lines still retain a considerable amount of maize’s genetic diversity, the historical dependence on a small set of ancestors has rendered the SS group more susceptible to the erosion of additive genetic variance over time. If not managed effectively, this vulnerability can lead to a decline in GCA within this group, ultimately restricting the genetic progress possible in inter-group hybrids in the long term (Goodman 2005).

A second challenge faced by maize breeders is the impracticability of evaluating all possible crosses between inbred lines, particularly in the early stages of a program. To address this limitation, several strategies have been developed to evaluate the performance of hybrids that have not yet been field-tested. An early approach was proposed by Bernardo (1994), who used restriction fragment length polymorphism markers to estimate genetic relationships among parental inbred lines and incorporated this information to predict the untested crosses. With the advent of high-density genotyping, this concept was expanded through genomic selection (GS), which exploits genome-wide marker data to model the genetic merit of individuals before any phenotypic evaluation (Meuwissen et al. 2001). In the context of maize breeding, GS enables breeders to assess the entire set of possible single-cross hybrids solely from parental genomic information. This capability, in turn, allows the identification of promising combinations and the filtering out of those with low potential without the need for extensive phenotypic testing (Technow et al. 2014; Kadam et al. 2016).

Among GS models, genomic best linear unbiased prediction (GBLUP) is one of the most widely applied in maize breeding (Cantelmo et al. 2017; Dalsente Krause et al. 2020). In its standard form, the GBLUP model relies on an additive relationship matrix derived from molecular markers to make predictions (VanRaden 2008). By accounting for realized genetic resemblance between individuals, GBLUP eliminates the bias that can occur in pedigree-based models, which assume that all close relatives share the same level of genetic relationship (Gamal El-Dien et al. 2016). Furthermore, GBLUP can be extended to multikernel models, in which nonadditive relationship matrices are incorporated alongside the additive matrix to also account for variation arising from dominance and epistasis effects (Su et al. 2012; Vitezica et al. 2013). The addition of these matrices can boost the model’s predictive ability and provide breeders with a deeper understanding of the genetic architecture of the traits being studied (Dias et al. 2018; Peixoto et al. 2024). These matrices also ensure an orthogonal separation of additive, dominance, and epistatic effects, which facilitates a clearer distinction between genetic variance components (Muñoz et al. 2014; Nazarian and Gezan 2016).

A set of 162 single-cross hybrids derived from SS and NSS inbred lines using a North Carolina Design II mating scheme (Comstock and Robinson 1948) was considered in this study. We developed GBLUP-based multikernel models using additive and nonadditive genomic relationship matrices to partition phenotypic variability into components associated with the GCA of both SS and NSS inbred lines, as well as variance attributable to SCA. For SCA effects, we evaluated two alternative nonadditive matrices: one based on dominance effects and another derived from additive genomic relationships among parental inbred lines. Moreover, we specified different variance–covariance structures to model genotype-by-environment interaction (GEI) effects explicitly. Using these models, we also predicted the performance of untested single-cross combinations across various training set configurations, which ranged from full parental representation to none. Our work addressed two fundamental aspects that could have major implications for maize breeding. First, we examined whether the SS and NSS parental inbred lines included in this study, which represent the long-established SS and NSS heterotic groups, still harbor sufficient GCA variance to sustain future gains. Second, we evaluated the extent to which the proposed GBLUP-based multikernel models improve predictive ability and provide insights into the genetic architecture of hybrid performance under varying levels of parental information.

## Materials and methods

### Genetic material

A diverse set of inbred lines from the two major maize heterotic groups of the US Corn Belt, SS and NSS, was assembled to serve as the parental pool for this study (Troyer 1999). The set consisted of 13 SS inbred lines and 28 NSS inbred lines, selected based on pedigree information, breeding history, geographic origin, and their relevance to the industry.

To maximize genetic diversity and ensure proper synchronization of flowering times, the inbred lines were categorized into early, intermediate, and late maturity groups (Table 1). Within each group, SS inbred lines (seed parent) were crossed with NSS inbred lines (pollen parent) to generate single-cross hybrids following a North Carolina Design II mating scheme (Comstock and Robinson 1948). Under this design, in which each seed parent was crossed with all pollen parents within a group, 72 early, 70 intermediate, and 75 late hybrids were theoretically expected. However, a total of 40, 67, and 74 hybrids were produced, respectively. The shortfall was primarily due to practical constraints, including flowering asynchrony and limited seed availability. Since some inbred lines were assigned to more than one maturity group, the total number of unique single-cross hybrids was 162, which is fewer than the total number of hybrids across the three groups (Supplementary Tables S1–S4).

**Table 1.** Stiff Stalk (SS) and Non-Stiff Stalk (NSS) inbred lines assigned to the early, intermediate, and late maturity groups according to their relative maturity classification.

Early		Intermediate		Late	
SS	NSS	SS	NSS	SS	NSS
LH145	PHR25	PHN66	PHG83	PHHB9	PHN47
CG120	PHN82	PHB47	Q381	PHV63	Q381
PHRE1	PHG29	PHW52	PHN82	PHP38	PHN82
PHB47	LH82	LH198	PHG29	LH195	PHW30
PHHV4	PHZ51	LH195	PHW30	PHW52	PHR63
S8324	LH185		LH82		PHM49
	PHK56		PHZ51		PHW53
	PHG47		PHR55		PHZ51
	LH162		LH185		PHR55
	CG123		PHK76		PHR03
	PHW03		LH38		LH185
	PHK05		PHK56		PHP60
			PHG47		LH210
			LH51		PHJ65
					PHM57

### Phenotypic data

As part of the Genomes to Fields (G2F) initiative (McFarland et al. 2020), the 162 single-cross hybrids were evaluated in multilocation field trials. In 2016, they were tested across 27 US locations and 2 Canadian locations, and in 2017, across 29 US locations and 2 Canadian locations. In both years, these hybrids were assessed alongside additional hybrids not included in this study but present in the same trials. These locations were grouped into three maturity sets, namely early, intermediate, and late, with an unbalanced distribution of individuals both within and between years. For analytical purposes, each unique combination of year and location was considered a distinct environment, resulting in a total of 60 environments. In each environment, trials were conducted using a randomized complete block design with two blocks, except in one instance where four blocks were used.

Although several agronomic traits were initially evaluated in the G2F trials, we concentrated on the following traits for our downstream analyses: grain yield (GY), reported in tons per hectare ($t\;ha^{-1}$); plant height (PH) and ear height (EH), measured in centimeters (cm); silking (SI), defined as the number of days from planting until silk emergence; and anthesis (AN), defined as the number of days from planting until pollen shed. These traits were assessed according to the standardized operating procedures established by the G2F initiative (Genomes to Fields Initiative 2025).

To ensure high-quality and consistent phenotypic information, we applied an initial round of filtering to the entire data set. This filtering included not only the hybrids of interest but also all hybrids evaluated in the G2F trials. Thus, the final data set after filtering included 806 maize hybrids and 44 environments. Importantly, none of the early, intermediate, and late single crosses needed to be removed during this process and are included among the 806 hybrids.

### Phenotypic analyses

#### Single-environment trial analyses

The following model was fitted to the entire filtered phenotypic data set to perform a single-environment trial analysis for each trait:


(1) y=μ1+Xb+Zh+e

where y (n×1) is the vector of phenotypic observations with *n* plots resulting from the combination of *g* hybrids and *r* blocks; *μ* is the intercept; b (r×1) is the vector of fixed effects of blocks; h (g×1) is the vector of random effects of hybrids, with $h∼\mathrm{MVN}(0,\;{I_{g}\sigma }_{h}^{2}$); and e (n×1) is the vector of random effects of residuals, with $e∼\mathrm{MVN}(0,\;I_{n}\sigma_{e}^{2})$. X (n×r) and Z (n×g) represent incidence matrices for their respective effects, 1 (n×1) denotes a vector of ones, $I_{g}$ and $I_{n}$ indicate identity matrices of appropriate dimensions, and $\sigma_{h}^{2}$ and $\sigma_{e}^{2}$ denote the variance components of hybrids and residuals, respectively. Based on this model, we calculated the broad-sense heritability ($H^{2}$) (Cullis et al. 2006) and the coefficient of variation (*CV*), as follows:


(2) $$H^{2}=1-\frac{\bar{PEV}}{2\hat{\sigma_{h}^{2}}}$$

(3) $$CV=\frac{\sqrt{\hat{\sigma_{e}^{2}}}}{\bar{y}}$$

where $\hat{\sigma_{h}^{2}}$ and $\hat{\sigma_{e}^{2}}$ are the estimated variance components of hybrids and residuals, respectively; $\bar{y}$ is the mean trait value within each environment; and $\bar{PEV}$ denotes the mean prediction error variance of the difference between two empirical best linear unbiased predictions (E-BLUPs) of the hybrid effects. The $\bar{PEV}$ represents the mean uncertainty associated with pairwise comparisons among hybrids and is therefore central to the definition of Cullis heritability (Cullis et al. 2006).

We also fitted a model similar to (1), this time treating hybrids as fixed effects, to obtain their empirical best linear unbiased estimations (E-BLUEs). Subsequently, we excluded environments with *CV* greater than 25% and/or those with near-zero $H^{2}$ from this set of E-BLUEs (Supplementary Table S5). In addition, we retained only the E-BLUEs corresponding to hybrids from the early, intermediate, and late single-cross groups. The resulting filtered data set of E-BLUEs for 162 single-cross hybrids at 37 environments was used as the response variable in all subsequent analyses. A summary of the phenotypic distributions and trait correlations, along with the co-occurrence matrix of hybrids across environments, is presented in Supplementary Figs. S1 and S2. Furthermore, Supplementary Table S6 provides the classification of these environments into maturity groups and their geographical distribution.

#### Multienvironment trial analyses

For each trait, we also performed a multienvironment trial analysis by fitting the following linear mixed model:


(4) $$y=\mu 1+X_{1}t\;+\;X_{2}h\;+\;Zht\;+\;e$$

where y (n×1) is the vector of E-BLUEs, obtained from the single-environment trial analyses (1), for *s* = 37 environments and *g* = 162 single-cross hybrids; $n=\sum_{i=1}^{s}n_{i}$, with $n_{i}$ being the number of single-cross hybrids in environment *i*; *μ* is the intercept; 1 (n×1) is a vector of ones; t (s×1) is the vector of fixed effects of environments; and h (g×1) is the vector of fixed effects of hybrids. The term ht (gs×1) represents the random interaction between hybrids and environments, modeled as $ht∼\mathrm{MVN}(0,G_{gs})$, where $G_{gs}=\;{(I}_{g}⊗\;D_{s}^{*})\sigma_{ht}^{2}$. Here, $I_{g}$ is an identity matrix of proper dimension, ⊗ denotes the Kronecker product, and $D_{s}^{*}$ is a diagonal matrix whose entries correspond to the environment-specific variance component, $\sigma_{ht}^{2}$, associated with the interaction effects. $X_{1}$ (n×s), $X_{2}$ (n×g), and Z (n×gs) are incidence matrices for their respective effects. In this framework, the variance--covariance matrix for the residual term e (n×1) was predefined as a diagonal matrix, with elements specified as:


(5) $$R_{n}=\left[\begin{array}{cccc} \frac{1}{\hat{SE_{1}^{2}}} & 0 & \cdots & 0\\ 0 & \frac{1}{\hat{SE_{2}^{2}}} & \cdots & 0\\ \vdots & \vdots & \ddots & \vdots \\ 0 & 0 & \cdots & \frac{1}{\hat{SE_{n}^{2}}} \end{array}\right]$$

where $\hat{SE}$ terms denote the standard errors derived from the single-trial analysis (Frensham et al. 1997). Using this multienvironmental model, we obtained E-BLUEs for hybrids, which were used in genomic prediction analyses.

### Genotypic data

The SS and NSS inbred lines belong to a broader panel of inbred lines from the G × E Project of the G2F initiative (Lima et al. 2023), which employed the Practical Haplotype Graph platform to generate variant calls (Bradbury et al. 2022). From this complete data set, we used VCFtools v0.1.15 (Danecek et al. 2011) to select only the individuals of interest, resulting in a subset of 41 inbred lines (13 SS and 28 NSS) and 437,214 SNPs.

For quality control, we used VCFtools v0.1.15 to filter out SNPs that were not bi-allelic, had a minor allele frequency below 5%, or exhibited more than 20% missing genotypes. We also removed redundant markers in high linkage disequilibrium (LD) using the LD pruning algorithm available in PLINK v1.9 (Purcell et al. 2007). This algorithm employed a sliding window approach to evaluate squared Pearson’s correlation coefficient ($r^{2}$) among SNPs within windows of 100 markers. When a pair of SNPs within a window displayed an $r^{2}$ value greater than 0.9, one of them was removed. The window was then advanced by 20 markers, and the procedure repeated. This approach preserved only one representative SNP from each highly correlated region, resulting in a final data set of 112,199 SNPs.

After applying quality control filters, we inferred the genotypes of all possible single-cross hybrids derived from the combinations between the 13 SS and 28 NSS parental inbred lines using TASSEL v5 (Bradbury et al. 2007). Finally, we converted the genotypic data for both the parental inbred lines and inferred hybrids into a numeric format using a custom R function. In this procedure, the most frequent allele at each locus was designated the reference allele, and genotypes were coded as 0 for minor homozygotes, 1 for heterozygotes, and 2 for major homozygotes, reflecting the number of copies of the reference allele.

### Genomic relationship matrices

Following the methodology proposed by VanRaden (2008), we computed the additive genomic relationship matrix (A) using the filtered and converted genotypic data of the 41 parental inbred lines, based on:


(6) $$A\;=\;\frac{ZZ^{⊤}}{2\sum_{\;j\;=1}^{m}\;p_{j}(1-p_{j})}$$

with:


$$Z=M-P=\left\{ \begin{array}{c} \begin{array}{c} 0-2p_{\;j}\\ \;1-2p_{\;j}\\ \;2-2p_{\;j} \end{array} \end{array}\right.$$

where M ($n_{g}\times m$) is the marker matrix of observed genotypes, coded as previously described; P ($n_{g}\times m$) is a matrix of expected genotype values, with each column representing a locus and being expressed as $P_{j}=2p_{j}$; $p_{j}$ is the allele frequency of the reference allele (ie the most frequent allele) at the *j*th locus; $n_{g}$ is the number of genotyped individuals; and *m* is the total number of markers. The matrix A, which represents the covariance structure for GCA effects, was then divided into $A_{SS}$, describing the relationships among the 13 SS inbred lines, and $A_{NSS}$, describing the relationships among the 28 NSS inbred lines.

To model the covariance structure of SCA effects in all possible single-cross hybrids derived from the combinations between the 13 SS and 28 NSS parental inbred lines, we investigated two types of relationship matrices. The first approach involved the construction of a dominance genomic relationship matrix (D) according to the method of Vitezica et al. (2013):


(7) $$D=\frac{WW^{⊤}}{\sum_{\;j=1}^{m}{\left({2p}_{j}(1-p_{j})\right)}^{2}}$$

with:


$$W=\left\{ \begin{array}{cc} -2p_{j}^{2} & \text{ for genotype coded as 0}\\ 2p_{j}(1-p_{j}) & \text{ for genotype coded as 1}\\ (1-p_{j}{)}^{2} & \text{ for genotype coded as 2} \end{array}\right.$$

where W ($n_{g}\times m$) is the dominance-coded marker matrix obtained by applying the above transformation to the entries of M at each locus *j*.

The second approach relied on a formulation described by Stuber and Cockerham (1966), which uses additive genetic relationships between parental inbred lines to construct a covariance matrix, referred to here as the matrix S. Specifically, for a given pair of single crosses, $H_{kl}={\mathrm{SS}}_{k}\times {\mathrm{NSS}}_{l}$ and $H_{k^{\prime }l^{\prime }}={\mathrm{SS}}_{k^{\prime }}\times {\mathrm{NSS}}_{l^{\prime }}$, the corresponding element of S was defined as:


(8) $$S[H_{kl},H_{k^{\prime }l^{\prime }}]=A_{SS}[{\mathrm{SS}}_{k},{\mathrm{SS}}_{k^{\prime }}]\cdot A_{NSS}[{\mathrm{NSS}}_{l},{\mathrm{NSS}}_{l^{\prime }}]$$

where ${\mathrm{SS}}_{k}$ and ${\mathrm{SS}}_{k^{\prime }}$ denote the *k*th and $k^{\prime }$th SS inbred lines, with $k,k^{\prime }\in 1,\ldots ,a$ and a=13. Similarly, ${\mathrm{NSS}}_{l}$ and ${\mathrm{NSS}}_{l^{\prime }}$ denote the *l*th and $l^{\prime th}$ NSS inbred lines, with $l,l^{\prime }\in 1,\ldots ,b$ and b=28. The terms $A_{SS}[{\mathrm{SS}}_{k},{\mathrm{SS}}_{k^{\prime }}]$ and $A_{NSS}[{\mathrm{NSS}}_{l},{\mathrm{NSS}}_{l^{\prime }}]$ correspond to the respective entries of the $A_{SS}$ and $A_{NSS}$ matrices, with the dot (·) indicating multiplication between them. Unlike D, which explicitly models dominance effects at the marker level, the S matrix does not explicitly capture dominance or epistatic interactions. Instead, it represents the expected covariance between single-cross hybrids based on the additive genetic relationships of their parental inbred lines. The distinct insights these matrices provide for modeling SCA effects will be discussed later.

The A and D matrices were calculated using the AGHmatrix R package v2.1.4 (Amadeu et al. 2016, 2023), whereas the S matrix was derived from a custom R function developed from the theoretical foundation described above. Because some of these matrices were not positive definite, their inverses were obtained using iterative bending methods, as implemented in the ASRgenomics package v1.1.4 (Gezan et al. 2022). Additionally, the similarity between D and S was quantified by computing the Pearson correlation between their off-diagonal elements.

### GBLUP-based multikernel models

#### Model specifications

A set of GBLUP-based multikernel models was fitted for each trait, with the model specified as:


(9) $$y=\mu 1+Xt+Z_{1}f+Z_{2}m+Z_{3}fm+Z_{4}ft+Z_{5}mt+Z_{6}fmt+e$$

where y (n×1) is the vector of E-BLUEs, obtained from the single-environment trial analyses (1), for *s* = 37 environments and *g* = 162 single-cross hybrids; $n=\sum_{i=1}^{s}n_{i}$, with $n_{i}$ being the number of single-cross hybrids in environment *i*; *μ* is the intercept; 1 (n×1) is a vector of ones; and t (s×1) is the vector of fixed effects of environments with incidence matrix X (n×s). The vectors f (a×1), m (b×1), and fm (ab×1) represent, respectively, the GCA effects of seed parents (SS inbred lines), the GCA effects of pollen parents (NSS inbred lines), and the SCA effects arising from their interaction, with corresponding incidence matrices $Z_{1}$ (n×a), $Z_{2}$ (n×b), and $Z_{3}$ (n×ab). The interactions between seed and pollen parents with environments are represented by the vectors ft (as×1) and mt (bs×1), along with their respective incidence matrices $Z_{4}$ (n×as) and $Z_{5}$ (n×bs). The vector fmt (abs×1), with incidence matrix $Z_{6}$ (n×abs), accounts for the three-way interaction among seed parents, pollen parents, and environments. All vectors f, m, fm, ft, mt, fmt, and the residual e were treated as random and assumed to be independent and normally distributed with zero means and variance–covariance matrices equal to:


$$\left[\begin{array}{c} f\\ m\\ fm\\ ft\\ mt\\ fmt\\ e \end{array}\right]∼\mathrm{MVN}\left(\left[\begin{array}{c} 0\\ 0\\ 0\\ 0\\ 0\\ 0\\ 0 \end{array}\right],\left[\begin{array}{ccccccc} G_{a}\sigma_{f}^{2} & 0 & 0 & 0 & 0 & 0 & 0\\ 0 & G_{b}\sigma_{m}^{2} & 0 & 0 & 0 & 0 & 0\\ 0 & 0 & G_{ab}\sigma_{\;fm}^{2} & 0 & 0 & 0 & 0\\ 0 & 0 & 0 & G_{as}\sigma_{\;ft}^{2} & 0 & 0 & 0\\ 0 & 0 & 0 & 0 & G_{bs}\sigma_{mt}^{2} & 0 & 0\\ 0 & 0 & 0 & 0 & 0 & G_{abs}\sigma_{\;fmt}^{2} & 0\\ 0 & 0 & 0 & 0 & 0 & 0 & R_{n} \end{array}\right]\right)$$

where each $\sigma^{2}$ term denotes the variance components of the random effects and $R_{n}$ denotes the residual variance—covariance matrix, as specified in (5). The variance—covariance matrices G were modeled using alternative structures, resulting in a collection of model configurations. The differences among these models pertain to the specification of $G_{ab}$, modeled using either the inverse of the D or the S matrix, and of $G_{as}$, $G_{bs}$, and $G_{abs}$, which were specified using either an identity matrix (I) or a diagonal matrix ($D_{s}^{*}$) (see Table 2).

**Table 2.** Summary of the six GBLUP-based multikernel models (Equation 9) considered for each trait, including the variance--covariance structures for parental effects ($G_{a}$ and $G_{b}$), their interaction ($G_{ab}$), and the genotype-by-environment interaction (GEI) effects ($G_{as}$, $G_{bs}$, and $G_{abs}$).

Model	Variance–covariance structure					
	$G_{a}$	$G_{b}$	$G_{ab}$	$G_{as}$	$G_{bs}$	$G_{abs}$
M1-D	$A_{SS}$	$A_{NSS}$	D	$I_{a}⊗I_{s}$	$I_{b}⊗I_{s}$	$I_{a}⊗I_{b}⊗I_{s}$
M2-D	$A_{SS}$	$A_{NSS}$	D	$I_{a}⊗I_{s}$	$I_{b}⊗I_{s}$	$I_{a}⊗I_{b}⊗D_{s}^{*}$
M3-D	$A_{SS}$	$A_{NSS}$	D	$I_{a}⊗D_{s}^{*}$	$I_{b}⊗D_{s}^{*}$	$I_{a}⊗I_{b}⊗D_{s}^{*}$
M1-S	$A_{SS}$	$A_{NSS}$	S	$I_{a}⊗I_{s}$	$I_{b}⊗I_{s}$	$I_{a}⊗I_{b}⊗I_{s}$
M2-S	$A_{SS}$	$A_{NSS}$	S	$I_{a}⊗I_{s}$	$I_{b}⊗I_{s}$	$I_{a}⊗I_{b}⊗D_{s}^{*}$
M3-S	$A_{SS}$	$A_{NSS}$	S	$I_{a}⊗D_{s}^{*}$	$I_{b}⊗D_{s}^{*}$	$I_{a}⊗I_{b}⊗D_{s}^{*}$

$A_{SS}$ : additive genomic relationship matrix for the Stiff Stalk (SS) inbred lines; $A_{NSS}$: additive genomic relationship matrix for the Non-Stiff Stalk (NSS) inbred lines; D: dominance genomic relationship matrix for single-cross hybrids derived from all possible crosses between parental inbred lines; S: covariance matrix derived from additive genetic relationships between parental inbred lines; $I_{a}$, $I_{b}$, $I_{s}$: identity matrices representing homogeneous variance across environments; $D_{s}^{*}$: diagonal matrix assuming a specific variance for each environment; and ⊗: Kronecker product.

The subscripts *a*, *b*, and *s* denote the number of seed parents (SS), pollen parents (NSS), and environments, respectively.

Model labels indicate the variance–covariance structure assumed for the genotype-byenvironment interaction (GEI) effects (M1–M3) and the relationship matrix used to model specific combining ability (SCA) effects (D or S).

In addition to the variance–covariance structures described above, we also explored factor-analytic (FA) mixed models of Smith et al. (2001) to estimate genetic correlations among environments and to model GEI. However, these models exhibited convergence difficulties and unstable variance components, with several variance components reaching zero. These results indicated that the data did not support a reliable FA-based representation of GEI, and therefore, we did not retain this approach for subsequent analyses.

To compare the models, we assessed their goodness of fit using the Akaike Information Criterion (AIC) (Akaike 1974), defined as:


(10) $$\mathrm{AIC}=-2\log\;L+2p_{m}$$

where *L* is the maximum point of the residual likelihood function and $p_{m}$ is the number of estimated parameters. Considering the alternative variance–covariance structures, we retained the model with the lowest AIC among those specified with the D matrix and, independently, the one with the lowest AIC among those specified with the S matrix. We also used diagnostic plots to identify potential outliers and evaluate the normality of residuals in the chosen models.

#### Estimation of genetic variance components

Using the best-fitting models defined by the D and S matrices (Table 2), we estimated the variance components associated with the GCA of parental inbred lines, as well as the variance components captured by the SCA of their interactions. Furthermore, we applied the best-fitting models separately for each maturity group to obtain group-specific variance component estimates. In these analyses, environments with limited representation of parental inbred lines and hybrids were excluded to minimize confounding effects, resulting in 18, 36, and 28 environments retained in the early, intermediate, and late groups, respectively.

To evaluate the significance of the variance components, we conducted likelihood ratio tests (LRTs), assuming a mixed chi-square distribution (Self and Liang 1987). In this process, each component was tested by comparing the full model with a reduced model in which the corresponding effect was removed. To further support the interpretation of the variance components, we evaluated the degree of independence among the GCA and SCA components, as proposed by Muñoz et al. (2014). For this purpose, we initially used the asymptotic variance–covariance matrix of these estimates to derive a correlation matrix and calculate its eigenvalues. Thereafter, we ordered the eigenvalues from largest to smallest and calculated the relative contribution of each eigenvalue to the total eigenvalue sum. Additionally, we calculated the cumulative proportions by sequentially summing the contributions of the ordered eigenvalues. Thus, the first cumulative proportion corresponds to the contribution of the largest eigenvalue alone, the second corresponds to the combined contribution of the two largest eigenvalues, and the third corresponds to the combined contribution of the three largest eigenvalues.

Under complete orthogonality, the correlation matrix approaches the identity matrix, resulting in eigenvalues of similar magnitude and equal relative contributions. In this scenario, the cumulative proportions follow a linear trend, whereas deviations from this pattern indicate departures from orthogonality among the variance components. So, by examining the cumulative proportions, we assessed whether the covariance structures defined by the $A_{SS}$, $A_{NSS}$, D, and S matrices behaved as expected with respect to orthogonality, thereby ensuring a reliable partitioning of the genetic variance components.

To facilitate interpretation of the variance partitioning, we also calculated the relative contribution of each component by measuring the ratio of its estimated variance to the total variance. For traits with interaction terms modeled under heterogeneous variance structures (see Table 2), we derived a single variance estimate by averaging across the weighted variance estimates, where each environment-specific variance was multiplied by the number of observations available in that environment. The resulting products were then summed across all environments, ensuring comparability with estimates obtained under homogeneous-variance assumptions. It is important to note that the relationship matrices described above were constructed using standardized parameterizations based on allele frequencies (VanRaden 2008; Vitezica et al. 2013). Therefore, all the resulting genetic variance components were expressed on a comparable scale and can be directly interpreted.

#### Genomic prediction of single-cross hybrids

We tested four methods to predict the performance of untested single-cross hybrids ($\hat{y_{u}}$) (Table 3). In the first two methods, predictions were generated either using only the GCA effects of the parental inbred lines or by combining GCA and SCA effects, as described by Technow et al. (2014) and Kadam et al. (2016):


(11) $$\hat{y_{u}}=\hat{\mu }+\hat{f}+\hat{m}$$

(12) $$\hat{y_{u}}=\hat{\mu }+\hat{f}+\hat{m}+\hat{fm}$$

where $\hat{\mu }$ is the estimated intercept; $\hat{f}$ and $\hat{m}$ are the predicted GCA effects associated with the SS and NSS parental inbred lines, respectively; and $\hat{fm}$ represents the predicted SCA effect of the untested single-cross hybrid. All terms were derived from the best-fitting GBLUP-based multikernel models specified with either the D or the S matrix (Table 2), resulting in variants 1a and 1b for Method 1 and variants 2a and 2b for Method 2 (Table 3). Although Method 1 relies solely on GCA effects, two variants were necessary because the prediction of these effects depended on the covariance structure used for SCA modeling.

**Table 3.** Genomic prediction methods used to infer the performance of untested single-cross hybrids.

Method	Variant	Prediction equation	Source of components
1	a	$\hat{y_{u}}=\hat{\mu }+\hat{f}+\hat{m}$	Multikernel model with D
	b	$\hat{y_{u}}=\hat{\mu }+\hat{f}+\hat{m}$	Multikernel model with S
2	a	$\hat{y_{u}}=\hat{\mu }+\hat{f}+\hat{m}+\hat{fm}$	Multikernel model with D
	b	$\hat{y_{u}}=\hat{\mu }+\hat{f}+\hat{m}+\hat{fm}$	Multikernel model with S
3	a	$\hat{y_{u}}\;=C_{ut}C_{tt}^{-1}\hat{y}$	$A_{SS}$ and $A_{NSS}$; multikernel model with D
	b	$\hat{y_{u}}\;=C_{ut}C_{tt}^{-1}\hat{y}$	$A_{SS}$ and $A_{NSS}$; multikernel model with S
4	a	$\hat{y_{u}}\;=C_{ut}C_{tt}^{-1}\hat{y}$	$A_{SS}$ , $A_{NSS}$, and D; multikernel model with D
	b	$\hat{y_{u}}\;=C_{ut}C_{tt}^{-1}\hat{y}$	$A_{SS}$ , $A_{NSS}$, and S; multikernel model with S

$\hat{y_{u}}$ : performance of untested single-cross hybrids; $\hat{\mu }$: intercept; $\hat{f}$: general combining ability (GCA) of seed parents (Stiff Stalk, SS, inbred lines); $\hat{m}$: GCA of pollen parents (Non-Stiff Stalk, NSS, inbred lines); $\hat{fm}$: specific combining ability (SCA) of single-cross hybrids between SS and NSS inbred lines; $C_{ut}$: genetic covariance matrix between untested and tested single-cross hybrids; $C_{tt}$: genetic covariance matrix among tested single-cross hybrids; $\hat{y}$: vector of E-BLUEs for the tested hybrids, obtained from Equation (4); $A_{SS}$: additive genomic relationship matrix for SS inbred lines; $A_{NSS}$: additive genomic relationship matrix for NSS inbred lines; D: dominance genomic relationship matrix for single-cross hybrids derived from all possible crosses between parental inbred lines; S: covariance matrix derived from additive genomic relationships between parental inbred lines.

The terms $\hat{f}$, $\hat{m}$, and $\hat{fm}$ were estimated from the best-fitting GBLUP-based multikernel model (Equation 9).

Method 1: untested hybrids were predicted from parental GCA effects only, using the GBLUP-based multikernel model (Equation 9) fitted with $A_{SS}$ and $A_{NSS}$, and either D (variant a) or S (variant b).

Method 2: as in Method 1, but including SCA effects.

Method 3: predictions were obtained from covariance matrices $C_{ut}$ and $C_{tt}$ constructed using $A_{SS}$ and $A_{NSS}$. In Method 3, variants differ in the source of variance components: (a) from the D-fitted model and (b) from the S-fitted model, both derived from the GBLUP-based multikernel model (Equation 9).

Method 4: as in Method 3, but including hybrid-level covariance through either D (variant 4a) or S (variant 4b), with variance components obtained from the corresponding best-fitting model (Equation 9).

Prediction equations correspond to Equations (11–13).

For comparison purposes, the last two methods were based on the covariance between tested and untested single-cross hybrids, as outlined in Bernardo (1996):


(13) $$\hat{y_{u}}=C_{ut}C_{tt}^{-1}\hat{y}$$

where $C_{ut}$ and $C_{tt}$ represent the genetic covariance matrices between untested and tested single crosses, and among tested single crosses, respectively, and $\hat{y}$ denotes the vector of E-BLUEs for the tested hybrids, obtained from Model (4). To illustrate how the covariance matrices $C_{ut}$ and $C_{tt}$ were constructed in each method, we indexed the parental SS inbred lines by $k,k^{\prime },k^{\prime \prime }\in \{1,\ldots ,a\}$, where a=13, and the parental NSS inbred lines by $l,l^{\prime },l^{\prime \prime }\in \{1,\ldots ,b\}$, where b=28, following the notation previously adopted for the construction of the S matrix. Additionally, we considered a hypothetical example involving three single-cross hybrids: $H_{kl}={\mathrm{SS}}_{k}\times {\mathrm{NSS}}_{l}$, $H_{k^{\prime }l^{\prime }}={\mathrm{SS}}_{k^{\prime }}\times {\mathrm{NSS}}_{l^{\prime }}$, and $H_{k^{\prime \prime }l^{\prime \prime }}={\mathrm{SS}}_{k^{\prime \prime }}\times {\mathrm{NSS}}_{l^{\prime \prime }}$. Here, $H_{kl}$ and $H_{k^{\prime }l^{\prime }}$ represent tested single-cross hybrids, whereas $H_{k^{\prime \prime }l^{\prime \prime }}$ denotes an untested single-cross hybrid. Accordingly, the elements of the $C_{ut}$ matrix in Method 3 were computed as:


(14) $$A_{SS}[{\mathrm{SS}}_{k^{\prime \prime }},{\mathrm{SS}}_{k^{*}}]\hat{\sigma_{f}^{2}}+A_{NSS}[{\mathrm{NSS}}_{l^{\prime \prime }},{\mathrm{NSS}}_{l^{*}}]\hat{\sigma_{m}^{2}}$$

where the bracketed element of $A_{SS}$ represents the additive genomic relationship between the SS parental inbred lines of the untested and tested single-cross hybrids, while the bracketed element of $A_{NSS}$ represents the corresponding additive genomic relationship between the NSS parental inbred lines. The indices $k^{*}$ and $l^{*}$ correspond to the parental inbred lines of either tested single-cross hybrid $H_{kl}$ or $H_{k^{\prime }l^{\prime }}$ in the hypothetical example, and the terms $\hat{\sigma_{f}^{2}}$ and $\hat{\sigma_{m}^{2}}$ denote the GCA variance components associated with the SS and NSS parental inbred lines, respectively. For the $C_{tt}$ matrix in Method 3, the off-diagonal elements were calculated as:


(15) $$A_{SS}[{\mathrm{SS}}_{k},{\mathrm{SS}}_{k^{\prime }}]\hat{\sigma_{f}^{2}}+A_{NSS}[{\mathrm{NSS}}_{l},{\mathrm{NSS}}_{l^{\prime }}]\hat{\sigma_{m}^{2}}$$

and the diagonal elements were calculated as:


(16) $$A_{SS}[{\mathrm{SS}}_{k^{*}},{\mathrm{SS}}_{k^{*}}]\hat{\sigma_{f}^{2}}+A_{NSS}[{\mathrm{NSS}}_{l^{*}},{\mathrm{NSS}}_{l^{*}}]\hat{\sigma_{m}^{2}}+\hat{\sigma_{se}^{2}}$$

where the bracketed element of $A_{SS}$ in Equation (15) represents the additive genomic relationship between the SS parental inbred lines of the tested single-cross hybrids $H_{kl}$ and $H_{k^{\prime }l^{\prime }}$, whereas the bracketed element of $A_{NSS}$ represents the corresponding additive genomic relationship between the NSS parental inbred lines. In Equation (16), the bracketed terms correspond to the diagonal elements of $A_{SS}$ and $A_{NSS}$ associated with the parental inbred lines of either of the tested single-cross hybrids $H_{kl}$ or $H_{k^{\prime }l^{\prime }}$ in the hypothetical example, and $\hat{\sigma_{se}^{2}}$ denotes the squared standard error associated with the E-BLUE of the corresponding hybrid, obtained from Model (4). In both equations, the variance components $\hat{\sigma_{f}^{2}}$ and $\hat{\sigma_{m}^{2}}$ are the same as those previously described in Equation (14). These variance components were also estimated from the best-fitting GBLUP-based multikernel models specified with either the D or the S matrix (Table 2), leading to variants 3a and 3b (Table 3). This distinction arises because all variance components are jointly estimated within a mixed model, and their values are influenced by the specification of the full covariance structure (Henderson 1975). In Method 4, these covariance matrices were further extended to incorporate hybrid-level information, with the elements of $C_{ut}$ computed as:


(17) $$A_{SS}[{\mathrm{SS}}_{k^{\prime \prime }},{\mathrm{SS}}_{k^{*}}]\hat{\sigma_{f}^{2}}+A_{NSS}[{\mathrm{NSS}}_{l^{\prime \prime }},{\mathrm{NSS}}_{l^{*}}]\hat{\sigma_{m}^{2}}+\left\{ \begin{array}{c} D[H_{k^{\prime \prime }l^{\prime \prime }},H_{k^{*}l^{*}}]\hat{\sigma_{\;fm}^{2}}\\ S[H_{k^{\prime \prime }l^{\prime \prime }},H_{k^{*}l^{*}}]\hat{\sigma_{\;fm}^{2}} \end{array}\right.$$

where the bracketed element of D represents the dominance genomic relationship between the untested and tested single-cross hybrids, the bracketed element of S represents the covariance between the untested and tested single-cross hybrids derived from the additive genomic relationships of their parental inbred lines, $H_{k^{*}l^{*}}$ denotes either of the tested single-cross hybrids in the hypothetical example, and $\hat{\sigma_{\;fm}^{2}}$ denotes the estimated SCA variance component obtained from the tested single-cross hybrids. All remaining terms retain the same meanings as previously described. For the $C_{tt}$ matrix, the off-diagonal elements of Method 4 were calculated as:


(18) $$A_{SS}[{\mathrm{SS}}_{k},{\mathrm{SS}}_{k^{\prime }}]\hat{\sigma_{f}^{2}}+A_{NSS}[{\mathrm{NSS}}_{l},{\mathrm{NSS}}_{l^{\prime }}]\hat{\sigma_{m}^{2}}+\left\{ \begin{array}{c} D[H_{kl},H_{k^{\prime }l^{\prime }}]\hat{\sigma_{\;fm}^{2}}\\ S[H_{kl},H_{k^{\prime }l^{\prime }}]\hat{\sigma_{\;fm}^{2}} \end{array}\right.$$

and the diagonal elements were calculated as:


(19) $$\begin{array}{rl} A_{SS}[{\mathrm{SS}}_{k^{*}},{\mathrm{SS}}_{k^{*}}]\hat{\sigma_{f}^{2}}+A_{NSS}[{\mathrm{NSS}}_{l^{*}},{\mathrm{NSS}}_{l^{*}}]\hat{\sigma_{m}^{2}} & +\left\{ \begin{array}{c} D[H_{k^{*}l^{*}},H_{k^{*}l^{*}}]\hat{\sigma_{\;fm}^{2}}\\ S[H_{k^{*}l^{*}},H_{k^{*}l^{*}}]\hat{\sigma_{\;fm}^{2}} \end{array}\right.\\ & +\hat{\sigma_{se}^{2}} \end{array}$$

where the bracketed elements of D and S in Equation (18) represent the relationships between tested single-cross hybrids. In contrast, in Equation (19), they represent the corresponding diagonal elements of the D and S matrices. The variance component $\hat{\sigma_{\;fm}^{2}}$ is the same as that presented in Equation (17) and was estimated from the best-fitting GBLUP-based multikernel models specified with either the D or the S matrix (Table 2). Therefore, when the third covariance component was modeled using D, the corresponding variance component was obtained from the model specified with D, whereas when modeled using S, the variance component was obtained from the model specified with S. As a result, modeling the third covariance component with either D or S produced variants 4a and 4b, respectively (Table 3). All other terms retain their previously described meanings.

#### Assessment of predictive abilities

The predictive ability of each method was calculated using leave-one-out cross-validation, in which each single-cross hybrid was sequentially removed from the data set to serve as the validation set, while the remaining data were used to train the model and predict the excluded hybrid (James et al. 2013). To evaluate the robustness of the predictive methods under varying levels of parental information, we tested four training set configurations: (i) T2, where both parents of the excluded hybrid were present in the training set through other hybrids; (ii) T1F, where all hybrids sharing the same seed parent (SS) were excluded; (iii) T1M, where all hybrids sharing the same pollen parent (NSS) were excluded; and (iv) T0, where all hybrids involving either parent of the validation hybrid were excluded, ensuring no direct parental information was available in the training set (Technow et al. 2014; Kadam et al. 2016). To avoid confounding effects due to variation in training set size, we determined the largest training set size common to all four prediction configurations, which was 125 hybrids. Following this criterion, we held out one hybrid as the validation unit and randomly sampled 125 hybrids without replacement from the remaining 161 to form the training set. This procedure was repeated 30 times to ensure adequate resampling.

Within each repetition and training set configuration, we fitted GBLUP-based multikernel models specified with either the D or the S matrix using the corresponding training set. From these models, we obtained the predicted GCA and SCA effects used in Methods 1 and 2, as well as the corresponding variance component estimates used to construct the covariance matrices in Methods 3 and 4. This procedure resulted in one predicted value per hybrid for each repetition, training set configuration, and method. For each repetition, we assessed predictive ability separately for each method and training set configuration by calculating the Pearson correlation between the E-BLUEs of the hybrids, computed from Model (4), and their genomic-predicted genotypic values. Finally, we assessed the overall predictive ability as the mean correlation across the 30 repetitions, along with the corresponding standard deviation to quantify variability.

### Model implementation

All linear models described in this study were fitted using the ASReml-R package v4 (The VSNi Team 2023). This package was particularly well-suited to our modeling framework because it allowed for the specification of complex variance--covariance structures for multilevel random effects. It also supported user-defined relationship matrices and facilitated the estimation of variance components using restricted maximum likelihood. Moreover, ASReml-R provided AIC values for model comparison and enabled LRT to assess the significance of individual variance components.

## Results

### The optimal variance–covariance structure was trait-dependent

The comparison of GBLUP-based multikernel models, summarized in Table 2, revealed clear differences in model fit across traits (Table 4). Among the models fitted with the dominance genomic relationship matrix (D), the one assuming environment-specific variance only for the three-way interaction term (M2-D) was favored for GY, EH, and SI. For PH, M1-D, which assumed homogeneous variances across environments, provided the best fit. In the case of AN, the preferred model was M3-D, which incorporated environment-specific variances for all interactions involving environments. Similarly, for the models based on the S matrix derived from additive genetic relationships between parental inbred lines, M1-S yielded the lowest AIC for PH, whereas M2-S was superior for GY, EH, and SI, and M3-S was selected for AN. These findings show that the ideal variance--covariance structure depends on the trait being examined. A homogeneous variance is adequate for PH, while heterogeneous structures are more suitable for GY, EH, SI, and AN.

**Table 4.** Akaike Information Criterion (AIC) values for GBLUP-based multikernel models (Equation 9) fitted under alternative variance–covariance structures by trait.

Model	AIC by trait				
	GY	PH	EH	SI	AN
M1-D	3,235.86	**13,137.95**	12,514.62	3,946.67	3,639.31
M2-D	**3,219.55**	13,166.43	**12,498.04**	**3,907.34**	3,621.63
M3-D	3,248.59	13,229.94	12,575.39	3,942.44	**3,606.75**
M1-S	3,234.32	**13,137.80**	12,512.74	3,947.54	3,642.29
M2-S	**3,218.15**	13,166.45	**12,496.24**	**3,907.80**	3,624.64
M3-S	3,247.29	13,229.68	12,573.55	3,943.04	**3,610.10**

GY: grain yield ($t\;ha^{-1}$), PH: plant height (cm), EH: ear height (cm), SI: silking (days), AN: anthesis (days).

Model labels indicate the variance–covariance structure assumed for the genotype-by-environment interaction (GEI) effects (M1–M3) and the relationship matrix used to model specific combining ability (SCA) effects (D or S) (see Table 2).

D : dominance genomic relationship matrix for single-cross hybrids derived from all possible crosses between parental inbred lines; S: covariance matrix derived from additive genetic relationships between parental inbred lines.

Lower AIC values indicate better model fit, and the best-fitting model for each trait was selected based on the minimum AIC.

Bold values indicate the lowest AIC.

As mentioned previously, the D matrix explicitly models dominance effects at the marker level, while the S matrix is based on additive genetic relationships between parental inbred lines. Despite these differences, both classes of models yielded similar AIC values and consistent rankings, suggesting that they captured very similar patterns of genetic relationship. This interpretation was further supported by the strong correlation observed between their off-diagonal elements, which was approximately 0.82.

### GCA variance drives genetic variation in both groups, with some notable exceptions

Dissecting the total phenotypic variance when all single-cross hybrids were analyzed together highlighted significant variability in both heterotic groups, confirming that SS and NSS inbred lines harbor GCA variance across all traits (Table 5). The contribution of NSS inbred lines to GCA variance was predominantly greater than that of SS inbred lines, particularly for PH and EH, where NSS explained nearly three times more variance. In contrast, the variance associated with SCA was smaller than that of the GCA components, indicating that SCA effects played a more limited role across the majority of traits under study. Regarding the D and S matrices, both parametrizations exhibited highly similar variance component patterns, consistent with previous evidence based on their comparable AIC values and the strong correlation between their off-diagonal elements. However, the S matrix produced smaller SCA estimates, suggesting that it captures these effects less directly. This difference is likely related to its construction, as it is not defined at the marker level but instead derived from parental relationships.

**Table 5.** Estimates of variance components associated with general combining ability (GCA) and specific combining ability (SCA), obtained from the best-fitting GBLUP-based multikernel model (Equation 9), for grain yield (GY, $t^{2}\;ha^{-2}$), plant height (PH, $cm^{2}$), ear height (EH, $cm^{2}$), silking (SI, $\mathrm{day}s^{2}$), and anthesis (AN, $\mathrm{day}s^{2}$).

Class^a^	Effect	Estimated variance components by trait				
		GY	PH	EH	SI	AN
D	SS	0.41	40.07	21.81	7.12	5.73
	NSS	0.92	135.22	59.87	10.80	9.98
	SS × NSS	0.31	14.57	5.82	0.47	0.57
S	SS	0.46	41.92	21.89	7.14	5.67
	NSS	0.88	130.84	58.31	10.85	10.04
	SS × NSS	0.07	3.28	1.30	0.11	0.13

SS: GCA of Stiff Stalk (SS) inbred lines used as seed parents, reflecting additive effects; NSS: GCA of Non-Stiff Stalk (NSS) inbred lines used as pollen parents, reflecting additive effects; and SS × NSS: SCA of single-cross hybrids between SS and NSS inbred lines, reflecting nonadditive effects.

^a^ This column indicates whether variance components were estimated from models specified with the D matrix (Class D) or with the S matrix (Class S).

D : dominance genomic relationship matrix for single-cross hybrids derived from all possible crosses between parental inbred lines; S: covariance matrix derived from additive genetic relationships between parental inbred lines.

When examining the variance components within each maturity group (Table 6), the overall pattern observed in the combined data set was mostly maintained, with a few noteworthy differences among the groups. In the early maturity set, the SCA component for EH was not significant. In the intermediate group, the most important maturity group for US maize production, a critical result emerged: the SS component for GY, the most economically relevant trait, was not significant. In this case, the variance from SCA was greater than that from SS, indicating that SS contributed less to the total phenotypic variance compared to other sources of variation in the intermediate group. For the SI and AN, the variances of the SS and NSS intermediate inbred lines were quite similar in magnitude, and SCA effects remained small. In the EH case, we found that the NSS variance was significant under the D model (59.34) but not under the S model (28.56). In the late maturity set, variance components were generally smaller than in the other groups, with some SCA estimates approaching zero. Nevertheless, several of these components remained statistically significant under the LRT, indicating that even minimal SCA effects were detectable. For further details on the standard errors of the estimates and the LRT statistics from the model comparisons, see Supplementary Tables S7–S10.

**Table 6.** Estimates of variance components associated with general combining ability (GCA) and specific combining ability (SCA) obtained from the best-fitting GBLUP-based multikernel model (Equation 9) within each maturity group for grain yield (GY, $t^{2}\;ha^{-2}$), plant height (PH, $cm^{2}$), ear height (EH, $cm^{2}$), silking (SI, $\mathrm{day}s^{2}$), and anthesis (AN, $\mathrm{day}s^{2}$).

Maturity group	Class^a^	Effect	GY	PH	EH	SI	AN
Early	D	SS	0.82	76.58	22.32	2.60	2.49
		NSS	1.49	106.28	49.76	6.35	5.56
		SS × NSS	0.18	2.20	0.71^ns^	1.11	1.29
	S	SS	0.76	79.46	23.05	2.93	2.83
		NSS	1.42	107.63	50.74	6.25	5.37
		SS × NSS	0.06	0.77	0.13^ns^	0.34	0.40
Intermediate	D	SS	0.02^ns^	7.19	23.16	3.92	2.97
		NSS	0.71	145.74	59.34	2.58	2.41
		SS × NSS	0.21	14.86	6.83	0.07	0.09
	S	SS	0.03^ns^	6.73	21.09	3.89	2.85
		NSS	0.30	113.37	28.56^ns^	2.22	2.05
		SS × NSS	0.15	8.06	4.93	0.06	0.07
Late	D	SS	0.11	7.90	3.39	0.75	0.75
		NSS	0.32	50.62	14.37	2.95	3.28
		SS × NSS	0.01	0.42	0.29	0.06	0.08
	S	SS	0.11	7.89	2.94	0.80	0.80
		NSS	0.31	50.89	13.88	2.69	3.05
		SS × NSS	0.01	0.40	0.39	0.05	0.06

SS: GCA of Stiff Stalk (SS) inbred lines used as seed parents, reflecting additive effects; NSS: GCA of Non-Stiff Stalk (NSS) inbred lines used as pollen parents, reflecting additive effects; SS × NSS: SCA of single-cross hybrids between SS and NSS inbred lines, reflecting nonadditive effects.

^a^ This column indicates whether variance components were estimated from models specified with the D matrix (Class D) or with the S matrix (Class S).

^ns^ Non-significant likelihood ratio test (p≥0.05).

D : dominance genomic relationship matrix for single-cross hybrids derived from all possible crosses between parental inbred lines; S: covariance matrix derived from additive genetic relationships between parental inbred lines.

**Fig. 1.**
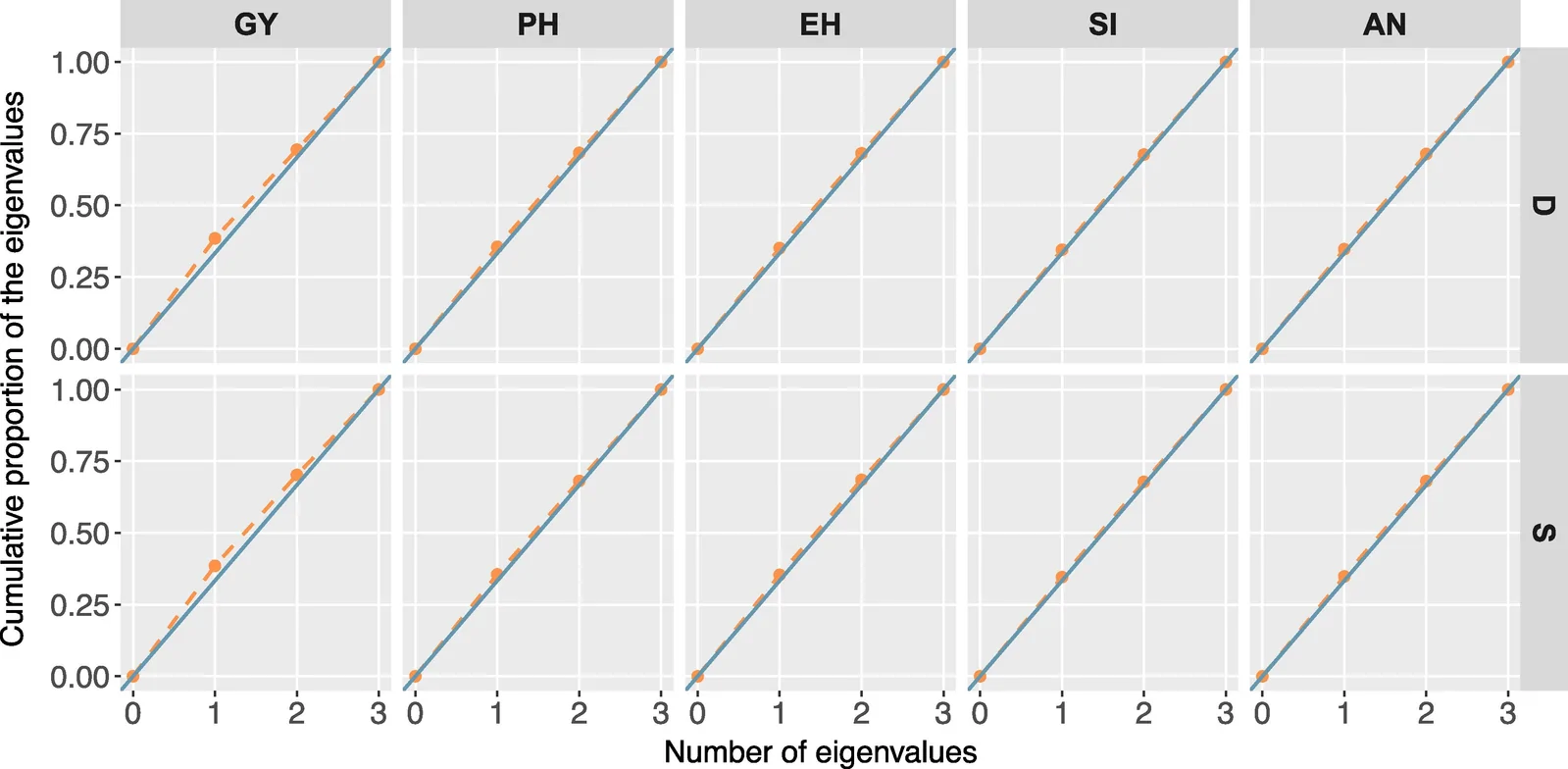
Cumulative proportion (dashed line) of the relative contribution of the ordered eigenvalues derived from the asymptotic correlation matrices of variance components associated with the general combining ability (GCA) of Stiff Stalk (SS) and Non-Stiff Stalk (NSS) inbred lines, and the specific combining ability (SCA) of single-cross hybrids between SS and NSS inbred lines, when all single-cross hybrids were analyzed together. The *x*-axis represents the rank of the eigenvalues, ordered from largest to smallest. The solid diagonal line represents the expected pattern under complete orthogonality (ie independence) among components, where all eigenvalues contribute equally and the cumulative proportion increases linearly. Deviations from this line indicate dependence among components. Results are shown for models fitted with either the D or S matrices in grain yield (GY, $t\;ha^{-1}$), plant height (PH, cm), ear height (EH, cm), silking (SI, days), and anthesis (AN, days). D: dominance genomic relationship matrix for single-cross hybrids derived from all possible crosses between parental inbred lines; S: covariance matrix derived from additive genetic relationships between parental inbred lines. The variance components were estimated from the GBLUP-based multikernel model described in Equation (9).

The degree of independence among the GCA and SCA components when all single-cross hybrids were analyzed together is illustrated in Fig. 1. Across all traits, the cumulative proportion of the eigenvalues closely follows the diagonal (solid blue line) under both the D and S parametrizations. This pattern indicates that the eigenvalues are nearly equal and that the corresponding components are nearly orthogonal. Consequently, the GCA effects of SS and NSS inbred lines, as well as the SCA effects, contribute independently to the total genetic variance, with minimal confounding among components. Therefore, the partitioning of genetic variance into its constituent components can be considered reliable, with no evidence of spurious dependencies among effects induced by the covariance structures.

**Fig. 2.**
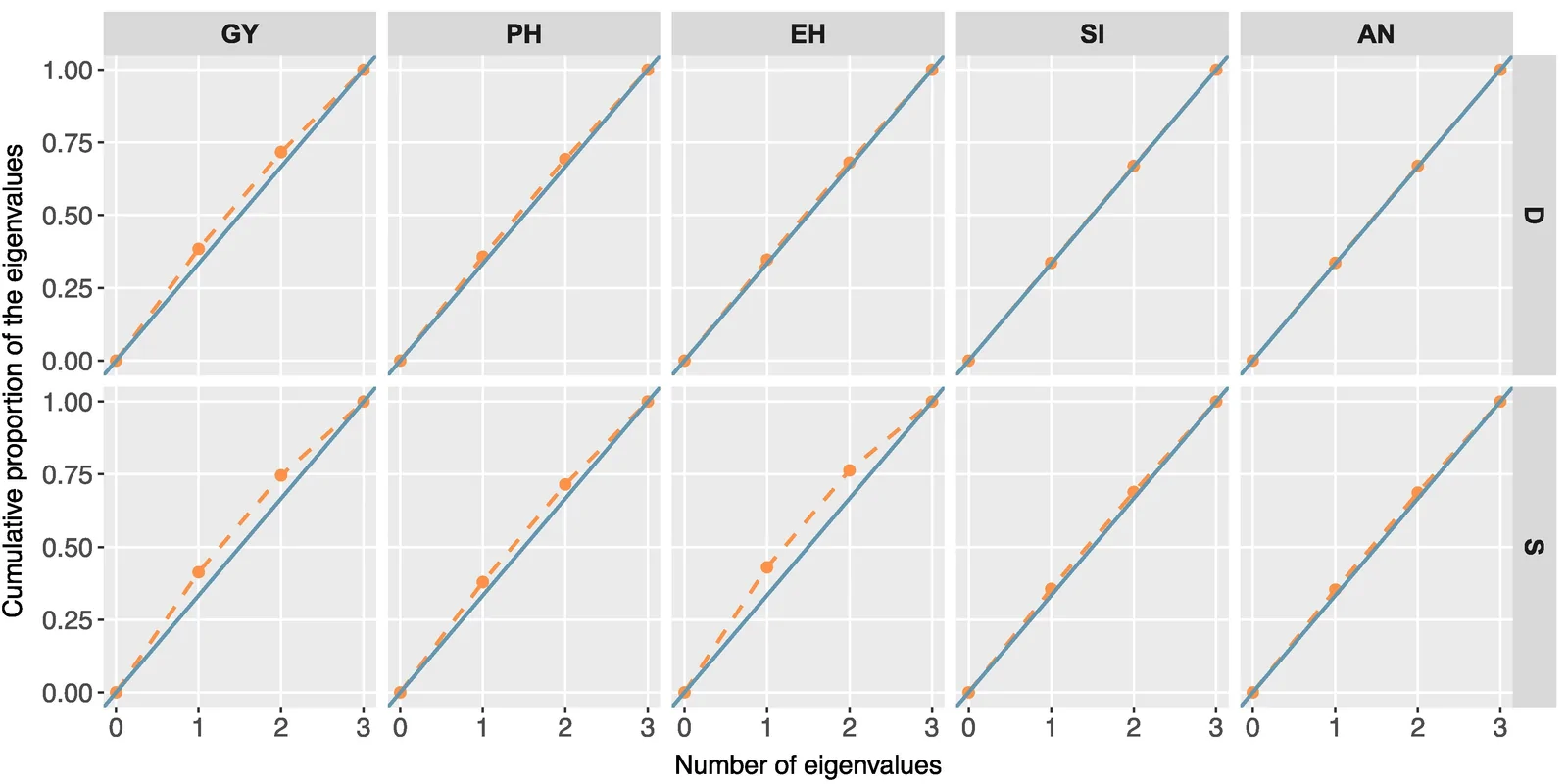
Cumulative proportion (dashed line) of the relative contribution of the ordered eigenvalues derived from the asymptotic correlation matrices of variance components associated with the general combining ability (GCA) of Stiff Stalk (SS) and Non-Stiff Stalk (NSS) inbred lines, and the specific combining ability (SCA) of single-cross hybrids between SS and NSS inbred lines, for the intermediate maturity group. The *x*-axis represents the rank of the eigenvalues, ordered from largest to smallest. The solid diagonal line represents the expected pattern under complete orthogonality (ie independence) among components, where all eigenvalues contribute equally and the cumulative proportion increases linearly. Deviations from this line indicate dependence among components. Results are shown for models fitted with either the D or S matrices in grain yield (GY, $t\;ha^{-1}$), plant height (PH, cm), ear height (EH, cm), silking (SI, days), and anthesis (AN, days). D: dominance genomic relationship matrix for single-cross hybrids derived from all possible crosses between parental inbred lines; S: covariance matrix derived from additive genetic relationships between parental inbred lines. The variance components were estimated from the GBLUP-based multikernel model described in Equation (9).

Separate analyses of the degree of independence between the GCA and SCA components, categorized by maturity group, demonstrated that the patterns observed for the early and late sets were in line with those found in the combined analysis. The cumulative proportion of the eigenvalues closely followed the diagonal in both parametrizations, indicating near-orthogonality among the components (see Supplementary Figs. S3 and S4). In contrast, the intermediate group revealed a distinct pattern (Fig. 2). For GY and, more markedly, for EH, the cumulative curve under the S parametrization deviated from the diagonal, suggesting that part of the variation attributed to SCA may be confounded with other components. This confounding likely reduces the statistical support for SCA, explaining why it was not significant under the S model, even though it was detected under the D parametrization (Table 6).

### SCA plays a major role in GEI in the intermediate and late maturity groups, despite the predominance of GCA

**Fig. 3.**
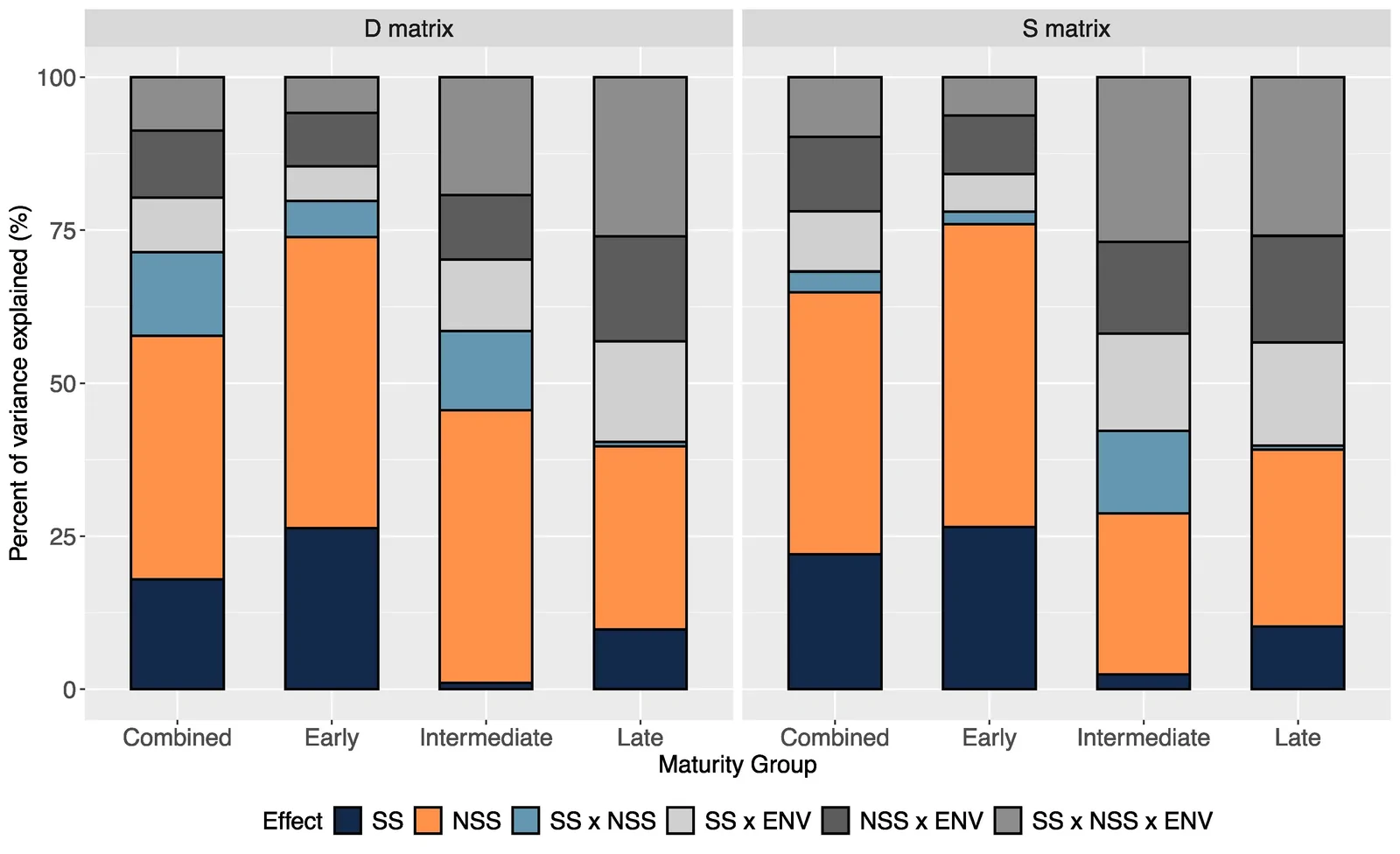
Percentage of variance explained for grain yield (GY, $t\;ha^{-1}$). Variance components were estimated from the GBLUP-based multikernel model described in Equation (9), using either the D or the S matrices. SS: general combining ability (GCA) of Stiff Stalk (SS) inbred lines used as seed parents, reflecting additive effects; NSS: GCA of Non-Stiff Stalk (NSS) inbred lines used as pollen parents, reflecting additive effects; SS × NSS: specific combining ability (SCA) of single-cross hybrids between SS and NSS inbred lines, reflecting nonadditive effects; SS × ENV: interaction between SS inbred lines and environments; NSS × ENV: interaction between NSS inbred lines and environments; SS × NSS × ENV: three-way interaction among SS, NSS, and environments, representing the interaction between SCA and environments; D: dominance genomic relationship matrix for single-cross hybrids derived from all possible crosses between parental inbred lines; S: covariance matrix derived from additive genetic relationships between parental inbred lines. Results are shown for all hybrids (combined) as well as separately by maturity group (early, intermediate, and late).

Figure 3 expands the variance partitioning by including the GEI terms, offering a more comprehensive view of the multiple sources of variation in GY. It should be noted that Fig. 3 does not include residual variance, as it was not re-estimated in Model (9) but rather predefined using weights derived from single-trial analyses. Consequently, the partitions presented here only reflect the genetic and interaction sources of variation, and the “total variance” needs to be understood as the sum of SS, NSS, SS × NSS, SS × ENV, NSS × ENV, and SS × NSS × ENV components. In the combined data set, which does not separate maturity groups, the GEI effects accounted for a non-negligible proportion of the explained variance across models specified with either the D or the S formulation. GEI represented about 25% of the explained variance, while the SS effect explained roughly 10% and the SS × NSS interaction about 15%.

By maturity groups, notable shifts in the proportion of variance explained by GEI became evident (Fig. 3). In early hybrids, nearly 70% of the observed variance was attributed to SS and NSS inbred lines, while GEI accounted for only a minor proportion, indicating that hybrid performance across environments was relatively stable. For intermediate hybrids, the proportion of variance explained by GEI increased compared with the early group. Notably, when considering all three interaction sources (SS × ENV, NSS × ENV, and SS × NSS × ENV), GEI accounted for more than 40% of the total variance. The highest percentage of GEI was observed in the late group, where interaction terms accounted for more than 50% of the total variance. The increase can largely be attributed to the broader environmental range. In our analysis of the raw data set, we found that late hybrids were grown not just in environments classified as late, but also in those categorized as early and intermediate. Such cross-testing likely accentuated differences in hybrid adaptation and consequently magnified GEI signals.

A closer examination of the interaction components revealed important differences in their relative contributions (Fig. 3). In both the intermediate and late maturity groups, the three-way interaction, SS × NSS × ENV, accounted for a substantially larger proportion of the variance than the interactions involving GCA (SS × ENV and NSS × ENV). As SS × NSS × ENV captures the interaction between SCA effects and environments, these results suggest that SCA effects were more responsive to environmental variation than GCA effects. Thus, the interaction between SCA and environments, alongside the GCA variance contributed by NSS inbred lines, played an important role in driving yield performance in this study.

Similar variance partitioning patterns were observed for the other traits (Supplementary Figs. S5–S8). Overall, the magnitude of GEI varied across traits, with some traits exhibiting a relatively small contribution of interaction effects, while others showed a substantial proportion of the total variance explained by GEI. In traits with higher GEI, the interaction components involving SCA (SS × NSS × ENV) tended to contribute more prominently, indicating a stronger environmental responsiveness of hybrid performance.

### Predictive ability hinges on parental information and the method used, with GCA effects providing sufficient accuracy in this study

**Fig. 4.**
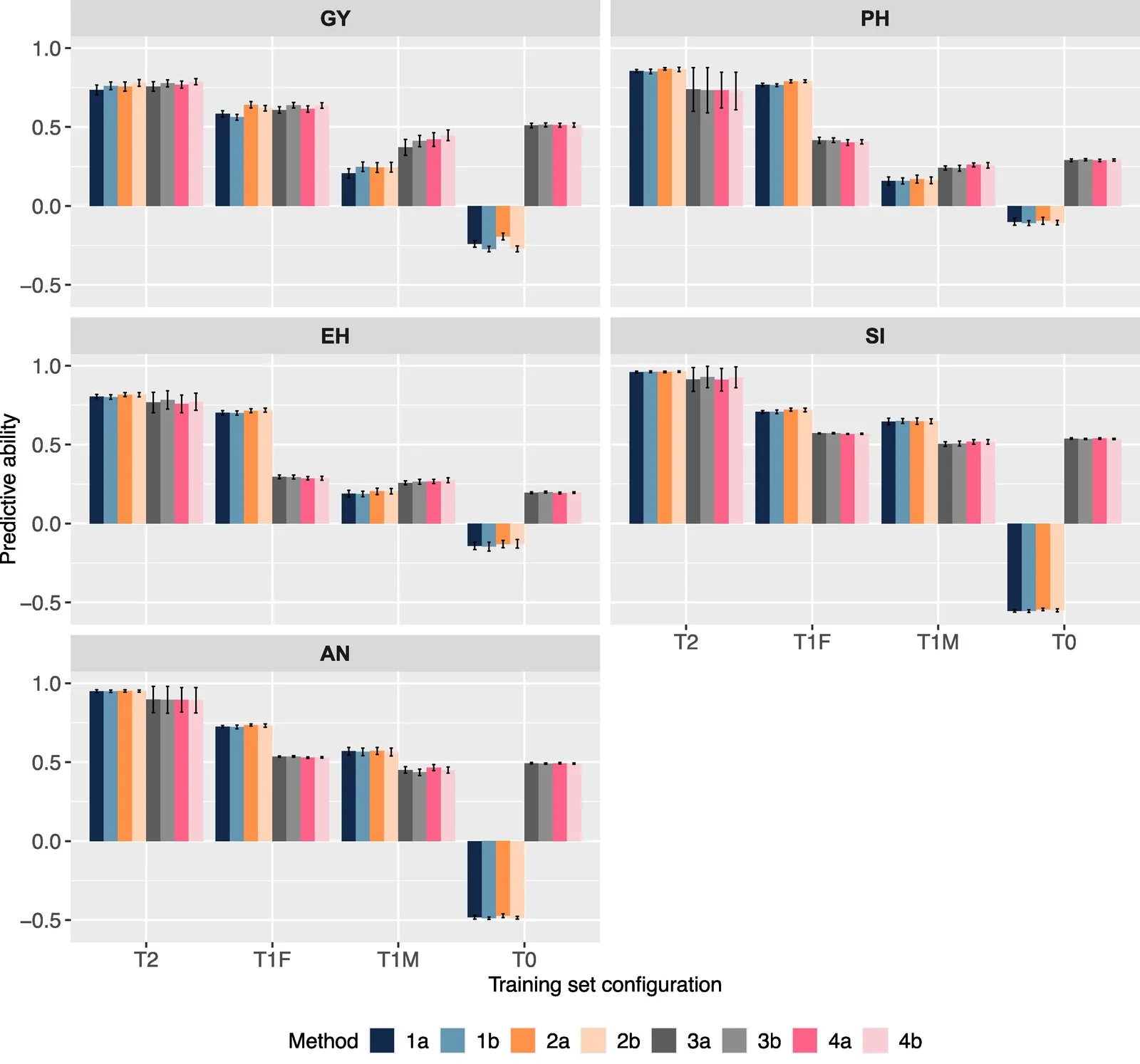
Predictive ability for untested single-cross hybrids for grain yield (GY, $t\;ha^{-1}$), plant height (PH, cm), ear height (EH, cm), silking (SI, days), and anthesis (AN, days), assessed under four training-set configurations: T2 (both parents of the validation hybrid represented via other crosses), T1F (all hybrids sharing the same seed parent excluded), T1M (all hybrids sharing the same pollen parent excluded), and T0 (no direct parental information in the training set). Methods 1–2 correspond to GBLUP-based multikernel models (Equation 9), where the first includes only general combining ability (GCA) (Equation 11) and the second includes both GCA and specific combining ability (SCA) (Equation 12). Methods 3–4 are based on the covariance between tested and untested single-cross hybrids (Equation 13), considering either the additive relationship matrix only or both additive and nonadditive relationship matrices. Results are shown for models fitted using either the D matrix (a) or the S matrix (b). D: dominance genomic relationship matrix for single-cross hybrids derived from all possible crosses between parental inbred lines; S: covariance matrix derived from additive genetic relationships between parental inbred lines. Additional model details are provided in Table 3.

Across traits, predictive ability declined as parental information was progressively removed from the training data (Fig. 4). Configuration T2, where training and validation sets shared the strongest connectedness, produced the highest correlations between E-BLUEs and genomic predicted genotypic values. In contrast, removing seed parents (SS) in T1F or pollen parents (NSS) in T1M resulted in a moderate decline in performance. The T0 configuration generally produced the lowest correlations, although its impact varied across traits and methods, with some combinations maintaining comparable levels to T1F and T1M. When considering individual traits, clear differences emerged. For GY, predictive abilities were the lowest across training set configurations and were strongly affected by the absence of parental information. For PH and EH, predictive abilities were intermediate relative to the other traits. Meanwhile, in line with their greater heritability (Supplementary Table S5), traits related to flowering time (SI and AN) showed the highest predictive ability when parental information was available. However, these traits also showed a pronounced decline under the T0 scenario, indicating a stronger dependence on parental information.

In comparing the methods that utilize GBLUP-based multikernel models (1a, 1b, 2a, and 2b) with those that focus on the covariance between tested and untested single-cross hybrids (3a, 3b, 4a, and 4b), notable differences were observed across traits and training configurations (Fig. 4). Under T2, methods using GBLUP-based multikernel models consistently outperformed covariance-based methods for most traits. The only exception to this trend was GY, where the predictive abilities of the two strategies were nearly overlapping. The T1F training set configuration followed the same general pattern as T2, but the advantage of GBLUP-based multikernel models became more pronounced, particularly for PH and EH, where the gap between the two approaches was larger. Results under T1M were more trait-dependent: GBLUP-based multikernel models showed superior performance for SI and AN, while covariance-based models achieved higher predictive abilities for PH, EH, and GY. The most contrasting outcomes were observed in the T0 scenario, which was the strictest. In this case, GBLUP-based multikernel models often yielded negative accuracy values across traits, particularly for GY, SI, and AN. In turn, the covariance-based methods continued to show higher predictive abilities for GY, SI, and AN, with moderate performance for PH and EH. This comparison clearly shows that GBLUP-based multikernel models generally perform better when parental information is available, particularly for traits with higher heritability, such as SI and AN. Conversely, covariance-based predictions tend to be more stable than GBLUP-based multikernel models in situations where direct parental representation is lacking.

The addition of SCA effects (2a–2b) or D and S matrices (4a–4b) produced negligible differences in predictive ability, indicating that the contribution of SCA effects played a limited role in this study (Fig. 4). These outcomes are consistent with the variance component estimates from the multikernel models in the combined analysis (Table 5 and Fig. 3), where GCA effects explained the largest portion of genetic variance across traits, particularly for PH, EH, SI, and AN in both SS and NSS heterotic groups. The predominance of GCA variance in these populations provided a natural rationale for the strong performance of models that rely on GCA to predict hybrid performance. Moreover, predictive abilities were nearly identical between variants that used the D matrix (a) and those that used the S matrix (b), also suggesting that these matrices capture highly similar patterns of genetic relationships among hybrids. This similarity was anticipated, as both parameterizations provided nearly identical variance components in most instances (Table 5). Detailed predictive ability results for each trait, including all methods and training set configurations, are provided in Supplementary Tables S11–S15.

## Discussion

The reduction of additive genetic variance within heterotic groups and the challenge of predicting untested single crosses at early stages remain central issues in maize breeding programs. Using GBLUP-based multikernel models, this study examined how GCA variance is distributed within SS and NSS germplasm and how parental information shapes predictive performance. The findings indicate that GCA remains a significant source of genetic signal for most traits. However, the study also highlights context-dependent limitations, particularly for GY in SS germplasm, and shows that predictive ability is strongly influenced by the degree of parental connectivity.

### Maintaining variance associated with GCA effects within heterotic groups is critical for sustained hybrid breeding progress

Additive variance refers to the portion of total genetic variance attributable to the average effects of alleles (Falconer 1996). It represents the effects that can be consistently transmitted from parents to progeny, and therefore it underlies the predictable response to selection (Lynch and Walsh 1998). In the classic breeder’s equation, the expected genetic gain is directly proportional to the amount of additive variance present in the population (Bernardo 2002). For this reason, the long-term success of maize hybrid breeding programs depends on maintaining additive variance within heterotic groups (Melchinger 1999; Hallauer et al. 2010). If this source of variance becomes depleted, further genetic gain cannot be achieved (Walsh and Lynch 2018).

The GCA effects of parental inbred lines capture differences in their average performance across multiple crosses, and these differences arise primarily from additive gene action. Based on this relationship, the magnitude of the GCA variance provides an indirect yet informative measure of the additive-related genetic variation available within each heterotic group. Thus, monitoring changes in GCA variance offers practical insight into the status and long-term sustainability of additive variance within breeding groups (Orton 2020).

The set of SS and NSS inbred lines analyzed in this study represents elite parental germplasm developed and released for hybrid breeding between the late 1980s and mid-1990s (Beckett et al. 2017). Over the decades that followed, repeated cycles of selection and the recycling of inbred lines within these heterotic groups have led to significant genetic progress. One clear example is the improvement in GY under drought-stress conditions (Gaffney et al. 2015). Recent analyses conducted in the US Corn Belt have shown that maize yields have increased in both favorable and stressed environments, averaging an annual gain of approximately 1.2%. Also, newer hybrids demonstrated improved drought resistance during the grain-filling period (Zhao et al. 2025). Nevertheless, this strategy of repeatedly drawing upon a relatively closed pool of parental inbred lines, combined with high selection intensities, increases the risk of allele fixation, reduces opportunities to discover new favorable combinations, and can lead to a gradual erosion of genetic diversity (Hallauer 1992; Fu 2015; Vieira et al. 2025).

Our findings demonstrate that, for the majority of traits evaluated, both SS and NSS heterotic groups have maintained significant GCA variance, whether analyzed jointly or within maturity groups. Similar findings were reported by Bornowski et al. (2021), who analyzed the SS germplasm using whole-genome assemblies and SNP-based diversity metrics, demonstrating that the pool retains significant genetic and genomic variation. Since their research focused on inbred lines, this residual diversity largely reflects the additive variance present within the population (Falconer 1996; Lynch and Walsh 1998). Our results, however, also highlight a critical concern: among the SS inbred lines represented in our data set, the GCA variance for GY in the intermediate maturity group was not statistically significant. This maturity group is crucial for the US Corn Belt, where intermediate hybrids dominate commercial fields and account for nearly one-third of global maize production (Zhao et al. 2025). Moreover, GY is the most important trait for the commercial success of maize. The lack of GCA variance for GY in intermediate inbred lines indicates a significant weakness in the SS germplasm, particularly in light of the increasing climatic challenges projected for the US Corn Belt (Zhao et al. 2025).

Looking forward, the concern extends beyond the intermediate set. The GCA variance still present in SS and NSS germplasm for other traits and maturity groups is a finite resource that, as discussed above, may gradually diminish over time (Hallauer 1992; Goodman 2005). To safeguard long-term progress, it is therefore essential not only to exploit the remaining variance but also to adopt strategies that prevent its depletion (Goodman 1999; Swarup et al. 2021; Salgotra and Chauhan 2023). Modern breeding tools, such as GS, play an important role in maintaining genetic diversity by limiting allele fixation and controlling relatedness within recurrent breeding programs (Li et al. 2022; Alemu et al. 2024). Complementary to these approaches, prebreeding efforts, like the Germplasm Enhancement of Maize project, have been instrumental in broadening the genetic base of commercial maize in the United States by incorporating novel and useful germplasm collected worldwide (Pollak and Salhuana 2000).

### The transient role of nonadditive variance captured by SCA in long-term genetic gain and the pervasive influence of GEI

Shull (1908) and East (1908) independently demonstrated that inbred maize lines exhibited reduced vigor and productivity, whereas crosses between distinct inbreds produced offspring with markedly improved performance due to a phenomenon later termed heterosis. Since then, unraveling the genetic basis of heterosis has been a major focus in maize research, with dominance, overdominance, and epistasis recognized as key mechanisms contributing to the superior performance of hybrids. In maize, these nonadditive mechanisms are not mutually exclusive and are captured by SCA effects (Garcia et al. 2025).

The magnitude of the SCA effects determines how much hybrid performance deviates from what is expected based on parental GCA effects alone (Hallauer 1992; Hallauer et al. 2010). In our study, the variance attributed to SCA was consistently smaller than the variance explained by GCA across traits. This finding aligns with the theoretical expectations of Reif et al. (2007), who showed that the ratio of dominance to additive variance decreases with increasing interpopulation divergence, resulting in a greater prevalence of GCA over SCA in divergent heterotic groups. A simulation study also demonstrated that both GCA and SCA variances tend to decrease across recurrent cycles of selection due to the strong selection intensity, with the reduction being particularly pronounced for SCA (Melchinger and Frisch 2023). These theoretical insights provide a compelling explanation for the lower magnitude of SCA variance observed in our data set and highlight that, while SCA contributes to the expression of heterosis, its role is transient compared with the more enduring additive effects in long-term genetic gain. Moreover, Melchinger and Frisch (2023) emphasized that the relative importance of SCA varies across traits, and our results are in line with this expectation: we found a higher SCA contribution for GY than for flowering-related traits, confirming that the expression of nonadditive variance is largely trait-dependent.

The GEI component represents a fundamental source of variation in hybrid breeding because it captures the differential response of genotypes across contrasting environments. Its presence complicates selection decisions, as superior performance in one environment does not necessarily translate into broad adaptation. Still, it also offers opportunities to exploit specific adaptation when target populations of environments are clearly defined (van Eeuwijk et al. 2016). Therefore, accounting for GEI is essential for accurate estimation of genetic effects and reliable prediction of hybrid performance, either via explicit interaction terms or variance–covariance structures that capture it implicitly. Numerous studies across maize have demonstrated that modeling GEI improves predictive ability and provides more realistic assessments of adaptation and stability, particularly for complex traits (Zhang et al. 2015; Dias et al. 2018).

The GEI component was modeled explicitly in this study, which allowed us to directly quantify its contribution to the total genetic variance. This approach revealed clear differences across maturity groups, with the most pronounced GEI detected in late hybrids. A likely explanation is that these environments included not only late-maturity hybrids but also early and intermediate ones, thereby amplifying differential responses and magnifying interaction effects. Historically, maturity groups have been central to US maize breeding because they align hybrids with the latitudinal gradient of production environments, ensuring that flowering and grain-filling occur under favorable seasonal conditions (Massigoge et al. 2023). The stronger GEI observed in late hybrids thus reflects both the broader environmental range in which they were tested and the intrinsic importance of maturity groups in structuring hybrid adaptation. These outcomes emphasize that explicitly accounting for GEI provides a more accurate understanding of the distribution of genetic variance and highlights its decisive role in shaping hybrid performance across environments. A more detailed inspection of the interaction components showed that the SCA effects were more sensitive to environmental variation than GCA effects in this study. A similar pattern was reported by Rogers et al. (2021) for GY. Although their study did not explicitly model SCA as in the present work, they showed that dominance effects contributed substantially to GEI and that GEI variance exceed genetic main effects, highlighting the strong environmental modulation of nonadditive genetic effects.

Given its importance in modeling GEI, an FA variance–covariance structure was initially considered in this study. The FA structure is a multivariate framework that captures the covariance among genetic effects across environments using a reduced number of latent factors (Smith et al. 2001). This method is widely used in multienvironment trials due to its efficient representation of GEI (Burgueño et al. 2012). However, in the present study, FA models exhibited convergence difficulties and boundary estimates, with several variance components constrained to zero. This pattern suggests overparameterization relative to the data structure, resulting in unstable covariance estimates and limiting the reliability of the FA-based representation of GEI (Smith et al. 2001; Kelly et al. 2007; Cullis et al. 2014). Consequently, we retained the explicit modeling of interaction terms while assuming independent environments with either homogeneous (identity) or heterogeneous (diagonal) variances. Although it does not account for genetic correlations among environments, this approach was better supported by the data and yielded more stable, interpretable results.

### Integrating mating designs and genomic relationship matrices for the orthogonal partition of genetic variance

In maize, dominance significantly influences hybrid performance, so modeling both additive and dominance effects is crucial for understanding trait architecture and guiding breeding strategies (Nazarian and Gezan 2016; Dias et al. 2018). One effective approach to achieve this is through suitable mating designs, such as the North Carolina design II, also known as a factorial design (Comstock and Robinson 1948). In this design, a set of pollen parents is systematically crossed with multiple seed parents, resulting in progenies that represent two distinct levels of relatedness: half-sib families, which come from either the pollen or seed parent, and full-sib families within each specific pollen-seed combination. Because both relationships are present, breeders can obtain complementary estimates of additive variance attributable to the parental inbred lines and dominance variance resulting from specific parental combinations (Bernardo 2002).

In scenarios where orthogonality is not ensured by design, molecular marker-based relationship matrices provide breeders with a robust framework for estimating genetic effects and their associated variance components (Bouvet et al. 2016; Dias et al. 2018; Nazarian and Gezan 2016). The additive genomic relationship matrix quantifies the proportion of alleles shared between individuals at each locus, whereas the dominance genomic relationship matrix reflects covariances due to specific allelic combinations at that locus. Although several methodologies can be used to generate these matrices, the most widely adopted are those proposed by VanRaden (2008) for additive effects and by Vitezica et al. (2013) and Muñoz et al. (2014) for dominance effects. In these approaches, the relationship matrices are centered and parameterized with respect to allele frequencies, ensuring that the expected covariance between additive and dominance components is zero. Consequently, additive and dominance effects are uncorrelated, enabling a near-orthogonal partitioning of genetic variance and a clearer separation of its underlying components.

Our results showed that the GCA effects of SS and NSS inbred lines, as well as the SCA effects, contributed largely independently to the total genetic variance, with minimal confounding among components. Although this near-orthogonality is expected under a factorial mating design, the use of ASS, ANSS, D, and S matrices provided an additional layer of robustness to this separation. In particular, these matrices offered a more accurate representation of genetic covariance among individuals, which is especially relevant in the presence of data imbalance or noise. Moreover, they allowed a practical framework for anticipating the performance of untested hybrids.

### Parental information and GCA variance are decisive for maize genomic prediction

The existence of a direct genetic relationship between the training and validation populations is fundamental to the success of genomic selection (Desta and Ortiz 2014; Ferrão et al. 2017), and this became evident in our study. As parental information was progressively removed from the training set, predictive ability declined, underscoring the central role of genetic connectedness in capturing the additive signal required for accurate predictions. This pattern, reflected in the superior performance of T2 over T1F, T1M, and especially T0, is consistent with previous research on maize hybrid prediction (Technow et al. 2014; Kadam et al. 2016). Using maize data-based simulations, Melchinger and Frisch (2023) also showed that prediction accuracy is maximized when more parents are represented in the training population, even if each contributes only a single cross. Another factor affecting GS accuracy is the amount of additive genetic variance (Desta and Ortiz 2014; Ferrão et al. 2017), which was highlighted by the sharper decline under T1M. In this scenario, the exclusion of NSS parents, who contributed most to this variance, markedly reduced predictive ability when compared to T1F.

GBLUP-based models (1a, 1b, 2a, and 2b) generally outperformed the covariance approaches (3a, 3b, 4a, and 4b), confirming their effectiveness when parental information was available. Under T0, however, they produced negative correlations for all traits, whereas the covariance approaches still yielded positive values. This discrepancy can be attributed to differences in how the two methods estimate effects. In GBLUP-based models, the GCA effects of individual parents and the SCA effects of their combinations are estimated using mixed-model equations (Henderson 1975). When neither parent of a hybrid is represented in the training set (T0), the model lacks anchors to predict these effects, which are consequently shrunk toward zero (Piepho et al. 2008). One possible explanation is that shrinkage of the estimates, combined with allele-frequency centering of genomic relationship matrices, may lead to negative predictive ability, even when genomic relationship matrices retain residual similarity (Piepho et al. 2008). In contrast, covariance-based approaches generate predictions by projecting the phenotypes of tested hybrids onto those of untested hybrids using covariance blocks ($\hat{y_{u}}=C_{ut}C_{tt}^{-1}\hat{y}$). In this framework, even if the exact parents of a hybrid are not represented in the training set, genomic similarity with other related parents or hybrids ensures nonzero covariance values. As a result, this information is directly incorporated into the predictions, resulting in stable, positive accuracies even in the absence of explicit parental overlap (Bernardo 1996).

Kadam et al. (2016) also evaluated genomic prediction under T0 conditions and reported positive accuracies. This contrast likely arises from differences in population structure. In their study, they utilized 312 hybrids derived from six biparental families, resulting in a highly interconnected dataset. Consequently, even when the exact parents of a hybrid were excluded, other hybrids with closely related parents were still represented, which probably sustained nonzero covariance and positive predictive ability. In our case, the diverse SS and NSS parental inbred lines produced a population with much lower indirect connectivity, making our T0 scenario more stringent.

Trait-specific differences in predictive ability reflected their underlying genetic architecture. Plant architectural and flowering traits such as PH, EH, SI, and AN showed higher accuracies because they are largely governed by additive genetic variance and exhibit higher heritability, allowing models to capture most of the genetic signal from parental information. By contrast, GY showed lower predictive ability and was disproportionately affected by the removal of parental connectivity, consistent with its smaller additive component and greater sensitivity to GEI and residual variance (Bernardo 2002). These results highlight the importance of modeling GEI in GS frameworks for complex traits such as GY, as failure to account for this source of variation can undermine prediction accuracy (Zhang et al. 2015; Dias et al. 2018).

The limited gains from including SCA effects in our models should not be interpreted as evidence that nonadditive effect is universally negligible. In quantitative genetics, the relevance of additive and nonadditive components always depends on the population and context in which they are defined (Lynch and Walsh 1998). For this germplasm, GCA variance clearly accounted for most of the observed phenotypic differences, which explains why models built only on GCA performed so well. However, other studies have shown meaningful increases in predictive ability when nonadditive effects were incorporated (Dias et al. 2018). This underscores the need for breeders to evaluate the genetic architecture of their target populations before deciding whether to include such terms, as the contribution of nonadditive effect is not a universal constant but a property of the specific genetic material under study (Garcia et al. 2025).

### D and S capture similar patterns but differ in biological interpretation

The D and S matrices showed strong similarity in their relationships, as indicated by the correlation between their off-diagonal elements. This pattern was further reflected in the highly consistent model fit, variance components, and predictive performance observed across traits. These results suggest that both matrices capture similar broad trends in the underlying genetic architecture, while also reinforcing the robustness of the parameterizations used in this study.

Although both matrices yield similar empirical results, the S matrix consistently produces smaller estimates of SCA variance, which can be explained by their distinct constructions. The S matrix is derived from additive genomic relationships between parental inbred lines and represents the expected covariance between hybrids based on parental similarity rather than their realized genotypic composition (Stuber and Cockerham 1966). While it may implicitly capture part of the nonadditive variation, including components arising from epistatic interactions, this representation remains indirect and averaged across loci. Moreover, the S matrix is defined as the product of parental relationships, which inherently reduces the scale of its values and induces a shrinkage effect. As a result, even though S may absorb part of the epistatic signal, this contribution is diluted within an expected covariance framework. In contrast, the D matrix is constructed from marker-level information and explicitly models dominance deviations at each locus, thereby capturing realized dominance effects with greater resolution and magnitude (Vitezica et al. 2013). Therefore, the choice between these parameterizations should not be viewed merely as a statistical alternative, but rather as a modeling decision that reflects different biological perspectives on the origin of SCA. Both parameterizations capture similar genetic signals but differ in how they quantify and interpret these signals.

## Conclusion

The GBLUP-based multikernel models developed in this study revealed that the SS and NSS parental inbred lines represented in our data set possess significant GCA variance, with NSS contributing more across various traits. However, the intermediate SS inbred lines, the most commercially relevant maturity group in the US Corn Belt, showed no significant GCA variance for GY. This putatively small amount of GCA variance limits the potential for sustained genetic improvement and underscores the need to expand the genetic base of SS germplasm to ensure long-term breeding progress. Furthermore, the GBLUP-based multikernel models performed well in T2, T1F, and T1M, where at least one parent was included in the training set, but poorly in T0, where no parental information was available. Since breeding programs typically operate under scenarios more comparable to T2, T1F, and T1M, our results demonstrate the practical value of this framework for improving the efficiency of maize hybrid breeding. Ultimately, although the D and S matrices used in this study differed in the magnitude of SCA variance, the strong agreement between their relationship structures indicated that they captured similar broad trends in the underlying genetic architecture.

## Supplementary Material

jkag163_Supplementary_Data

## Data Availability

All scripts and processed data supporting this study are publicly available via Zenodo at https://doi.org/10.5281/zenodo.19713122. The Zenodo record is linked to the corresponding GitHub repository, which includes documentation and instructions to retrieve the raw phenotypic and genotypic data from the G2F Data Commons (CyVerse). Supplemental material available at G3 online.
