# Supplementary material for: Dissecting genetic variance structure and evaluating genomic prediction models for single-cross hybrids derived from Stiff Stalk and Non-Stiff Stalk maize heterotic groups
Source: G3 (Bethesda). 2026 Jul 3;16(9):jkag163. doi: 10.1093/g3journal/jkag163 (PMC13535482; doi:10.1093/g3journal/jkag163)
Supplement: Supplementary file 1 [file jkag163_supplementary_data.pdf]

Supplementary Information

Dissecting genetic variance structure and evaluating genomic prediction models for single-cross hybrids derived from Stiff Stalk and Non-Stiff Stalk maize heterotic groups

Supplementary Table S1: List of single-cross hybrids derived from Stiff Stalk (SS) × Non-Stiff Stalk (NSS) inbred lines that were exclusively assigned to the early maturity group.

SS	NSS	SS × NSS	Maturity Group
LH145	PHR25	LH145/PHR25	Early
CG120	PHR25	CG120/PHR25	Early
PHRE1	PHR25	PHRE1/PHR25	Early
PHB47	PHR25	PHB47/PHR25	Early
PHHV4	PHR25	PHHV4/PHR25	Early
S8324	PHR25	S8324/PHR25	Early
CG120	PHZ51	CG120/PHZ51	Early
PHHV4	PHZ51	PHHV4/PHZ51	Early
S8324	PHZ51	S8324/PHZ51	Early
CG120	LH185	CG120/LH185	Early
PHHV4	LH185	PHHV4/LH185	Early
PHHV4	PHK56	PHHV4/PHK56	Early
S8324	PHK56	S8324/PHK56	Early
LH145	LH162	LH145/LH162	Early
CG120	LH162	CG120/LH162	Early
PHRE1	LH162	PHRE1/LH162	Early
PHB47	LH162	PHB47/LH162	Early
PHHV4	LH162	PHHV4/LH162	Early
S8324	LH162	S8324/LH162	Early
LH145	CG123	LH145/CG123	Early
CG120	CG123	CG120/CG123	Early
PHRE1	CG123	PHRE1/CG123	Early
PHB47	CG123	PHB47/CG123	Early
PHHV4	CG123	PHHV4/CG123	Early
S8324	CG123	S8324/CG123	Early
CG120	PHW03	CG120/PHW03	Early
PHB47	PHW03	PHB47/PHW03	Early
PHHV4	PHW03	PHHV4/PHW03	Early
LH145	PHK05	LH145/PHK05	Early
CG120	PHK05	CG120/PHK05	Early
PHB47	PHK05	PHB47/PHK05	Early
PHHV4	PHK05	PHHV4/PHK05	Early
S8324	PHK05	S8324/PHK05	Early

Supplementary Table S2: List of single-cross hybrids derived from Stiff Stalk (SS) × Non-Stiff Stalk (NSS) inbred lines that were exclusively assigned to the intermediate maturity group.

SS	NSS	SS × NSS	Maturity Group
PHN66	PHG83	PHN66/PHG83	Intermediate
PHB47	PHG83	PHB47/PHG83	Intermediate
PHW52	PHG83	PHW52/PHG83	Intermediate
LH198	PHG83	LH198/PHG83	Intermediate
LH195	PHG83	LH195/PHG83	Intermediate
PHN66	Q381	PHN66/Q381	Intermediate
PHB47	Q381	PHB47/Q381	Intermediate
LH198	Q381	LH198/Q381	Intermediate
PHN66	PHN82	PHN66/PHN82	Intermediate
LH198	PHN82	LH198/PHN82	Intermediate
PHN66	PHG29	PHN66/PHG29	Intermediate
PHW52	PHG29	PHW52/PHG29	Intermediate
LH198	PHG29	LH198/PHG29	Intermediate
LH195	PHG29	LH195/PHG29	Intermediate
PHN66	LH82	PHN66/LH82	Intermediate
PHW52	LH82	PHW52/LH82	Intermediate
LH198	LH82	LH198/LH82	Intermediate
LH195	LH82	LH195/LH82	Intermediate
PHN66	PHZ51	PHN66/PHZ51	Intermediate
LH198	PHZ51	LH198/PHZ51	Intermediate
PHN66	PHR55	PHN66/PHR55	Intermediate
PHB47	PHR55	PHB47/PHR55	Intermediate
LH198	PHR55	LH198/PHR55	Intermediate
PHN66	LH185	PHN66/LH185	Intermediate
LH198	LH185	LH198/LH185	Intermediate
PHN66	PHK76	PHN66/PHK76	Intermediate
PHB47	PHK76	PHB47/PHK76	Intermediate
PHW52	PHK76	PHW52/PHK76	Intermediate
LH198	PHK76	LH198/PHK76	Intermediate
LH195	PHK76	LH195/PHK76	Intermediate
PHN66	LH38	PHN66/LH38	Intermediate
PHB47	LH38	PHB47/LH38	Intermediate
PHW52	LH38	PHW52/LH38	Intermediate
LH198	LH38	LH198/LH38	Intermediate
LH195	LH38	LH195/LH38	Intermediate
PHN66	PHK56	PHN66/PHK56	Intermediate
PHW52	PHK56	PHW52/PHK56	Intermediate
LH198	PHK56	LH198/PHK56	Intermediate
LH195	PHK56	LH195/PHK56	Intermediate
PHN66	PHG47	PHN66/PHG47	Intermediate
PHW52	PHG47	PHW52/PHG47	Intermediate
LH198	PHG47	LH198/PHG47	Intermediate
LH195	PHG47	LH195/PHG47	Intermediate
PHN66	LH51	PHN66/LH51	Intermediate
PHB47	LH51	PHB47/LH51	Intermediate
PHW52	LH51	PHW52/LH51	Intermediate
LH198	LH51	LH198/LH51	Intermediate
LH195	LH51	LH195/LH51	Intermediate

Supplementary Table S3: List of single-cross hybrids derived from Stiff Stalk (SS) × Non-Stiff Stalk (NSS) inbred lines that were exclusively assigned to the late maturity group.

SS	NSS	SS × NSS	Maturity Group
PHHB9	PHN47	PHHB9/PHN47	Late
PHV63	PHN47	PHV63/PHN47	Late
PHP38	PHN47	PHP38/PHN47	Late
LH195	PHN47	LH195/PHN47	Late
PHW52	PHN47	PHW52/PHN47	Late
PHHB9	Q381	PHHB9/Q381	Late
PHV63	Q381	PHV63/Q381	Late
PHP38	Q381	PHP38/Q381	Late
PHHB9	PHN82	PHHB9/PHN82	Late
PHV63	PHN82	PHV63/PHN82	Late
PHP38	PHN82	PHP38/PHN82	Late
PHHB9	PHW30	PHHB9/PHW30	Late
PHV63	PHW30	PHV63/PHW30	Late
PHP38	PHW30	PHP38/PHW30	Late
PHHB9	PHR63	PHHB9/PHR63	Late
PHV63	PHR63	PHV63/PHR63	Late
PHP38	PHR63	PHP38/PHR63	Late
LH195	PHR63	LH195/PHR63	Late
PHW52	PHR63	PHW52/PHR63	Late
PHHB9	PHM49	PHHB9/PHM49	Late
PHV63	PHM49	PHV63/PHM49	Late
PHP38	PHM49	PHP38/PHM49	Late
LH195	PHM49	LH195/PHM49	Late
PHW52	PHM49	PHW52/PHM49	Late
PHHB9	PHW53	PHHB9/PHW53	Late
PHV63	PHW53	PHV63/PHW53	Late
PHP38	PHW53	PHP38/PHW53	Late
LH195	PHW53	LH195/PHW53	Late
PHW52	PHW53	PHW52/PHW53	Late
PHHB9	PHZ51	PHHB9/PHZ51	Late
PHV63	PHZ51	PHV63/PHZ51	Late
PHHB9	PHR55	PHHB9/PHR55	Late
PHV63	PHR55	PHV63/PHR55	Late
PHP38	PHR55	PHP38/PHR55	Late
PHHB9	PHR03	PHHB9/PHR03	Late
PHV63	PHR03	PHV63/PHR03	Late
PHP38	PHR03	PHP38/PHR03	Late
LH195	PHR03	LH195/PHR03	Late
PHW52	PHR03	PHW52/PHR03	Late
PHHB9	LH185	PHHB9/LH185	Late
PHV63	LH185	PHV63/LH185	Late
PHP38	LH185	PHP38/LH185	Late
PHHB9	PHP60	PHHB9/PHP60	Late
PHV63	PHP60	PHV63/PHP60	Late
PHP38	PHP60	PHP38/PHP60	Late
LH195	PHP60	LH195/PHP60	Late
PHW52	PHP60	PHW52/PHP60	Late
PHHB9	LH210	PHHB9/LH210	Late
PHV63	LH210	PHV63/LH210	Late
PHP38	LH210	PHP38/LH210	Late
LH195	LH210	LH195/LH210	Late
PHW52	LH210	PHW52/LH210	Late
PHHB9	PHJ65	PHHB9/PHJ65	Late
PHV63	PHJ65	PHV63/PHJ65	Late
PHP38	PHJ65	PHP38/PHJ65	Late
LH195	PHJ65	LH195/PHJ65	Late
PHW52	PHJ65	PHW52/PHJ65	Late
PHHB9	PHM57	PHHB9/PHM57	Late
PHV63	PHM57	PHV63/PHM57	Late
PHP38	PHM57	PHP38/PHM57	Late
LH195	PHM57	LH195/PHM57	Late
PHW52	PHM57	PHW52/PHM57	Late

Supplementary Table S4: List of single-cross hybrids derived from Stiff Stalk (SS) $\times$ Non-Stiff Stalk (NSS) inbred lines that were simultaneously assigned to more than one maturity group.

SS	NSS	SS $\times$ NSS	Maturity Group
PHB47	PHN82	PHB47/PHN82	Early/Intermediate
PHB47	PHG29	PHB47/PHG29	Early/Intermediate
PHB47	LH82	PHB47/LH82	Early/Intermediate
PHB47	PHZ51	PHB47/PHZ51	Early/Intermediate
PHB47	LH185	PHB47/LH185	Early/Intermediate
PHB47	PHK56	PHB47/PHK56	Early/Intermediate
PHB47	PHG47	PHB47/PHG47	Early/Intermediate
PHW52	Q381	PHW52/Q381	Intermediate/Late
LH195	Q381	LH195/Q381	Intermediate/Late
PHW52	PHN82	PHW52/PHN82	Intermediate/Late
LH195	PHN82	LH195/PHN82	Intermediate/Late
PHW52	PHW30	PHW52/PHW30	Intermediate/Late
LH195	PHW30	LH195/PHW30	Intermediate/Late
PHW52	PHZ51	PHW52/PHZ51	Intermediate/Late
LH195	PHZ51	LH195/PHZ51	Intermediate/Late
PHW52	PHR55	PHW52/PHR55	Intermediate/Late
LH195	PHR55	LH195/PHR55	Intermediate/Late
PHW52	LH185	PHW52/LH185	Intermediate/Late
LH195	LH185	LH195/LH185	Intermediate/Late

Supplementary Table S5: Broad-sense heritability (H^2) and coefficient of variation (CV, %) for grain yield (GY, t ha^{-1}), plant height (PH, cm), ear height (EH, cm), silking (SI, days), and anthesis (AN, days) across environments retained for analysis, i.e., those not excluded due to excessively high CV (>25%) or near-zero H^2 values.

Environment	GY		PH		EH		SI		AN	
	H^2	CV	H^2	CV	H^2	CV	H^2	CV	H^2	CV
2016_ARH1	0.61	13.29	0.66	7.03	0.29	13.02	0.92	1.65	0.92	1.49
2016_ARH2	0.39	21.77	0.50	6.66	0.37	12.00	0.88	1.99	0.87	1.96
2016_DEH1	0.76	13.90	0.70	5.09	0.64	11.27	0.57	2.38	0.59	2.27
2016_GAH1	0.60	18.82	0.69	6.81	0.67	10.40	0.89	2.05	0.89	1.98
2016_IAH2	0.80	16.25	0.29	7.05	0.38	12.27	0.92	1.63	0.93	1.50
2016_IAH3	0.26	17.48	0.31	6.25	0.16	12.23	0.63	2.76	0.60	2.34
2016_IAH4	0.58	12.91	0.75	6.09	0.76	10.67	0.90	2.12	0.92	1.58
2016_INH1	0.71	18.32	0.71	5.41	0.68	8.74	0.65	3.02	0.74	2.65
2016_MIH1	0.68	17.71	0.71	6.37	0.74	9.53	0.94	1.79	0.90	2.28
2016_MOH1	0.63	16.90	0.82	3.90	0.70	8.14	0.88	2.06	0.89	1.75
2016_NEH1	0.70	13.78	0.70	3.33	0.61	11.91	0.90	0.87	0.68	1.11
2016_NEH4	0.38	15.88	0.87	2.58	0.86	7.20	0.67	1.86	0.63	1.49
2016_NYH1	0.56	13.88	0.66	8.46	0.71	11.54	0.86	2.37	0.92	1.97
2016_ONH1	0.57	12.49	0.47	10.35	0.56	11.52	0.87	2.22	0.96	1.18
2016_ONH2	0.63	14.68	0.80	4.89	0.73	10.38	0.97	1.48	0.98	1.37
2016_TXH1	0.75	12.33	0.72	4.79	0.46	17.72	0.89	1.42	0.89	1.48
2016_TXH2	0.61	16.91	0.53	6.22	0.68	8.49	0.90	1.64	0.91	1.63
2016_WIH1	0.72	14.69	0.31	6.82	0.39	11.45	0.93	1.54	0.91	1.65
2016_WIH2	0.73	19.67	0.87	3.23	0.84	5.68	0.94	1.68	0.94	1.51
2017_COH1	0.69	19.53	0.81	5.90	0.79	11.51	0.96	2.71	0.97	2.41
2017_DEH1	0.80	11.37	0.83	5.73	0.74	9.39	0.70	3.95	0.70	3.75
2017_IAH4	0.75	11.01	0.90	4.54	0.87	8.21	0.96	1.68	0.97	1.23
2017_INH1	0.68	10.55	0.88	3.24	0.77	7.58	0.85	1.96	0.87	1.80
2017_MIH1	0.58	17.12	0.69	4.32	0.68	7.70	0.71	3.72	0.76	3.75
2017_MNH1	0.31	21.25	0.32	9.63	0.33	17.57	0.81	3.25	0.82	3.22
2017_MOH1	0.42	18.09	0.92	3.67	0.87	7.55	0.91	1.64	0.92	1.46
2017_NYH1	0.63	17.58	0.74	4.68	0.79	8.83	0.93	2.07	0.92	1.84
2017_NYH2	0.39	15.32	0.76	4.70	0.80	8.01	0.94	1.86	0.95	1.66
2017_NYH3	0.48	17.60	0.94	3.63	0.94	5.42	0.86	2.21	0.87	1.89
2017_ONH1	0.55	14.23	0.58	4.80	0.12	14.09	0.82	2.35	0.83	2.22
2017_ONH2	0.54	10.98	0.45	7.45	0.57	13.30	0.74	7.69	0.71	7.17
2017_TXH1-Dry	0.80	7.93	0.78	3.10	0.68	5.59	0.92	1.32	0.92	1.30
2017_TXH1-Early	0.84	6.77	0.78	3.33	0.72	6.01	0.91	1.19	0.89	1.36
2017_TXH1-Late	0.70	12.14	0.88	3.45	0.68	9.58	0.88	1.67	0.86	1.80
2017_TXH2	0.63	17.41	0.49	8.18	0.68	8.85	0.70	2.56	0.66	2.70
2017_WIH1	0.73	12.72	0.79	4.95	0.76	9.44	0.86	2.73	0.87	2.60
2017_WIH2	0.64	11.20	0.94	3.66	0.90	7.31	0.96	1.22	0.96	1.31

Supplementary Table S6: Classification into maturity groups and geographical distribution of the 37 environments retained for analysis.

Environment	Year	Location	Country	Maturity Group
2016_ARH1	2016	ARH1	USA	Late
2016_ARH2	2016	ARH2	USA	Late
2016_DEH1	2016	DEH1	USA	Intermediate
2016_GAH1	2016	GAH1	USA	Late
2016_IAH2	2016	IAH2	USA	Early/Intermediate
2016_IAH3	2016	IAH3	USA	Intermediate/Late
2016_IAH4	2016	IAH4	USA	Early/Intermediate
2016_INH1	2016	INH1	USA	Intermediate
2016_MIH1	2016	MIH1	USA	Early
2016_MOH1	2016	MOH1	USA	Intermediate
2016_NEH1	2016	NEH1	USA	Intermediate
2016_NEH4	2016	NEH4	USA	Intermediate
2016_NYH1	2016	NYH1	USA	Early
2016_ONH1	2016	ONH1	Canada	Early
2016_ONH2	2016	ONH2	Canada	Early
2016_TXH1	2016	TXH1	USA	Late
2016_TXH2	2016	TXH2	USA	Late
2016_WIH1	2016	WIH1	USA	Early/Intermediate
2016_WIH2	2016	WIH2	USA	Early/Intermediate
2017_COH1	2017	COH1	USA	Early/Intermediate
2017_DEH1	2017	DEH1	USA	Intermediate
2017_IAH4	2017	IAH4	USA	Early/Intermediate
2017_INH1	2017	INH1	USA	Intermediate
2017_MIH1	2017	MIH1	USA	Early
2017_MNH1	2017	MNH1	USA	Early
2017_MOH1	2017	MOH1	USA	Intermediate
2017_NYH1	2017	NYH1	USA	Early
2017_NYH2	2017	NYH2	USA	Early
2017_NYH3	2017	NYH3	USA	Intermediate
2017_ONH1	2017	ONH1	Canada	Early
2017_ONH2	2017	ONH2	Canada	Early
2017_TXH1-Dry	2017	TXH1-Dry	USA	Late
2017_TXH1-Early	2017	TXH1-Early	USA	Late
2017_TXH1-Late	2017	TXH1-Late	USA	Late
2017_TXH2	2017	TXH2	USA	Late
2017_WIH1	2017	WIH1	USA	Early/Intermediate
2017_WIH2	2017	WIH2	USA	Early/Intermediate

Supplementary Table S7: Estimates of variance components associated with general combining ability (GCA) and specific combining ability (SCA), along with standard errors for grain yield (GY, t^2 ha^{-2}), plant height (PH, cm^2), ear height (EH, cm^2), silking (SI, $days^2$), and anthesis (AN, $days^2$), considering all maturity groups combined. Likelihood ratio test (LRT) statistics and *p*-values are reported for each variance component.

Trait	Class[1]	Effect	Variance Component	Standard Error	LRT-statistic	*p*-value[2]
GY	D	SS	0.41	0.24	10.05	7.61e-04
		NSS	0.92	0.31	52.65	1.99e-13
		SS × NSS	0.31	0.05	192.69	2.20e-16
	S	SS	0.46	0.25	15.76	3.59e-05
		NSS	0.88	0.32	36.83	6.43e-10
		SS × NSS	0.07	0.01	194.08	2.20e-16
PH	D	SS	40.07	19.63	44.58	1.22e-11
		NSS	135.22	40.45	120.28	2.20e-16
		SS × NSS	14.57	2.59	189.07	2.20e-16
	S	SS	41.92	20.07	56.21	3.26e-14
		NSS	130.84	39.87	94.40	2.20e-16
		SS × NSS	3.28	0.58	189.21	2.20e-16
EH	D	SS	21.81	10.20	59.90	4.99e-15
		NSS	59.87	18.10	89.25	2.20e-16
		SS × NSS	5.83	1.31	63.88	6.66e-16
	S	SS	21.89	10.09	73.43	2.20e-16
		NSS	58.31	18.01	68.69	2.20e-16
		SS × NSS	1.30	0.29	65.67	2.78e-16
SI	D	SS	7.12	3.05	124.61	2.20e-16
		NSS	10.80	3.07	158.46	2.20e-16
		SS × NSS	0.47	0.08	192.99	2.20e-16
	S	SS	7.14	3.04	155.60	2.20e-16
		NSS	10.85	3.11	135.30	2.20e-16
		SS × NSS	0.11	0.02	192.52	2.20e-16
AN	D	SS	5.73	2.48	117.25	2.20e-16
		NSS	9.98	2.85	154.68	2.20e-16
		SS × NSS	0.57	0.09	271.10	2.20e-16
	S	SS	5.67	2.43	145.55	2.20e-16
		NSS	10.04	2.90	126.91	2.20e-16
		SS × NSS	0.13	0.02	267.75	2.20e-16

SS: GCA of Stiff Stalk (SS) inbred lines used as seed parents, reflecting additive effects; NSS: GCA of Non-Stiff Stalk (NSS) inbred lines used as pollen parents, reflecting additive effects; and SS × NSS: SCA of single-cross hybrids between SS and NSS inbred lines, reflecting dominance effects.

[1]This column indicates whether variance components were estimated from models specified with the **D** matrix (Class D) or with the **S** matrix (Class S).

[2]Presented *p*-values are given in scientific notation. All variance components were statistically significant (*p*-value < 0.001).

[ns] Non-significant likelihood ratio test ($p \geq 0.05$).

D: dominance genomic relationship matrix for single-cross hybrids derived from all possible crosses between parental inbred lines; **S**: covariance matrix derived from additive genetic relationships between parental inbred lines.

Estimates of variance components were obtained from the best-fitting GBLUP-based multi-kernel model (Equation 9).

Supplementary Table S8: Estimates of variance components associated with general combining ability (GCA) and specific combining ability (SCA), along with standard errors for the early maturity group, focusing on grain yield (GY, t^2 ha^{-2}), plant height (PH, cm^2), ear height (EH, cm^2), silking (SI, $days^2$), and anthesis (AN, $days^2$). Likelihood ratio test (LRT) statistics and *p*-values are reported for each variance component.

Trait	Class[1]	Effect	Variance Component	Standard Error	LRT-statistic	*p*-value[2]
GY	D	SS	0.82	0.60	15.93	3.29e-05
		NSS	1.49	0.76	20.51	2.97e-06
		SS × NSS	0.18	0.07	46.87	3.79e-12
	S	SS	0.76	0.55	16.54	2.38e-05
		NSS	1.42	0.73	16.93	1.94e-05
		SS × NSS	0.06	0.02	51.29	4.00e-13
PH	D	SS	76.58	50.86	38.33	2.98e-10
		NSS	106.28	49.28	44.37	1.36e-11
		SS × NSS	2.20	1.36	6.13	6.66e-03
	S	SS	79.46	52.60	49.02	1.27e-12
		NSS	107.63	49.90	42.31	3.89e-11
		SS × NSS	0.77	0.47	7.62	2.88e-03
EH	D	SS	22.32	15.30	25.99	1.71e-07
		NSS	49.76	23.52	37.46	4.68e-10
		SS × NSS	0.71	0.81	0.93	0.17^{ns}
	S	SS	23.05	15.64	38.60	2.61e-10
		NSS	50.74	23.79	36.77	6.65e-10
		SS × NSS	0.13	0.27	0.17	0.34^{ns}
SI	D	SS	2.60	1.99	10.69	5.38e-04
		NSS	6.35	3.26	19.38	5.34e-06
		SS × NSS	1.11	0.35	95.39	2.20e-16
	S	SS	2.93	2.15	14.57	6.75e-05
		NSS	6.25	3.17	17.52	1.42e-05
		SS × NSS	0.34	0.11	99.32	2.20e-16
AN	D	SS	2.49	1.95	9.74	9.00e-04
		NSS	5.56	2.93	17.62	1.35e-05
		SS × NSS	1.29	0.39	186.69	2.20e-16
	S	SS	2.83	2.09	15.37	4.43e-05
		NSS	5.37	2.81	15.81	3.51e-05
		SS × NSS	0.40	0.12	190.60	2.20e-16

SS: GCA of Stiff Stalk (SS) inbred lines used as seed parents, reflecting additive effects; NSS: GCA of Non-Stiff Stalk (NSS) inbred lines used as pollen parents, reflecting additive effects; and SS × NSS: SCA of single-cross hybrids between SS and NSS inbred lines, reflecting dominance effects.

[1]This column indicates whether variance components were estimated from models specified with the **D** matrix (Class D) or with the **S** matrix (Class S).

[2]Presented *p*-values are given in scientific notation. Variance components showed different levels of significance, including $p < 0.01$ and $p < 0.001$. Cells marked with ^{ns} indicate non-significant effects ($p \geq 0.05$).

D: dominance genomic relationship matrix for single-cross hybrids derived from all possible crosses between parental inbred lines; **S**: covariance matrix derived from additive genetic relationships between parental inbred lines.

Estimates of variance components were obtained from the best-fitting GBLUP-based multi-kernel model (Equation 9).

Supplementary Table S9: Estimates of variance components associated with general combining ability (GCA) and specific combining ability (SCA), along with standard errors for the intermediate maturity group, focusing on grain yield (GY, t^2 ha^{-2}), plant height (PH, cm^2), ear height (EH, cm^2), silking (SI, $days^2$), and anthesis (AN, $days^2$). Likelihood ratio test (LRT) statistics and p-values are reported for each variance component.

Trait	Class[1]	Effect	Variance Component	Standard Error	LRT-statistic	p-value[2]
GY	D	SS	0.02	0.04	0.22	0.32^{ns}
		NSS	0.71	0.33	24.55	3.62e-07
		SS × NSS	0.21	0.05	160.68	2.20e-16
	S	SS	0.03	0.05	0.66	0.21^{ns}
		NSS	0.30	0.23	4.77	0.02
		SS × NSS	0.15	0.04	177.01	2.20e-16
PH	D	SS	7.19	7.12	4.22	2.00e-02
		NSS	145.74	61.72	53.64	1.20e-13
		SS × NSS	14.86	3.52	137.17	2.20e-16
	S	SS	6.73	7.13	4.92	1.32e-02
		NSS	113.37	55.06	13.54	1.17e-04
		SS × NSS	8.06	2.03	167.68	2.20e-16
EH	D	SS	23.16	17.48	30.75	1.47e-08
		NSS	59.34	25.64	44.47	1.29e-11
		SS × NSS	6.83	1.80	56.91	2.28e-14
	S	SS	21.09	16.92	11.03	4.49e-04
		NSS	28.56	18.44	0.00	0.50^{ns}
		SS × NSS	4.93	1.39	79.22	2.20e-16
SI	D	SS	3.92	2.80	138.45	2.20e-16
		NSS	2.58	1.05	100.35	2.20e-16
		SS × NSS	0.07	0.02	45.59	7.29e-12
	S	SS	3.89	2.80	90.13	2.20e-16
		NSS	2.22	0.96	45.22	8.80e-12
		SS × NSS	0.06	0.02	58.35	1.09e-14
AN	D	SS	2.97	2.13	118.89	2.20e-16
		NSS	2.41	0.98	93.55	2.20e-16
		SS × NSS	0.09	0.02	74.54	2.20e-16
	S	SS	2.85	2.06	79.46	2.20e-16
		NSS	2.05	0.89	46.96	3.62e-12
		SS × NSS	0.07	0.02	89.20	2.20e-16

SS: GCA of Stiff Stalk (SS) inbred lines used as seed parents, reflecting additive effects; NSS: GCA of Non-Stiff Stalk (NSS) inbred lines used as pollen parents, reflecting additive effects; and SS × NSS: SCA of single-cross hybrids between SS and NSS inbred lines, reflecting dominance effects.

[1]This column indicates whether variance components were estimated from models specified with the **D** matrix (Class D) or with the **S** matrix (Class S).

[2]Presented p-values are given in scientific notation. Variance components showed different levels of significance, including $p < 0.05$ and $p < 0.001$. Cells marked with ^{ns} indicate non-significant effects ($p \geq 0.05$).

D: dominance genomic relationship matrix for single-cross hybrids derived from all possible crosses between parental inbred lines; **S**: covariance matrix derived from additive genetic relationships between parental inbred lines.

Estimates of variance components were obtained from the best-fitting GBLUP-based multi-kernel model (Equation 9).

Supplementary Table S10: Estimates of variance components associated with general combining ability (GCA) and specific combining ability (SCA), along with standard errors for the late maturity group, focusing on grain yield (GY, t^2 ha^{-2}), plant height (PH, cm^2), ear height (EH, cm^2), silking (SI, $days^2$), and anthesis (AN, $days^2$). Likelihood ratio test (LRT) statistics and p-values are reported for each variance component.

Trait	Class[1]	Effect	Variance Component	Standard Error	LRT-statistic	p-value[2]
GY	D	SS	0.11	0.09	6.40	5.71e-03
		NSS	0.32	0.14	59.76	5.39e-15
		SS × NSS	0.01	0.00	5.19	1.14e-02
	S	SS	0.11	0.09	6.38	0.01
		NSS	0.31	0.14	40.34	1.07e-10
		SS × NSS	0.01	0.00	7.70	2.77e-03
PH	D	SS	7.90	6.72	7.65	2.83e-03
		NSS	50.62	20.83	118.24	2.20e-16
		SS × NSS	0.42	0.22	7.60	2.92e-03
	S	SS	7.89	6.81	7.18	3.68e-03
		NSS	50.89	21.21	70.18	2.20e-16
		SS × NSS	0.40	0.22	5.68	8.58e-03
EH	D	SS	3.39	2.53	34.68	1.94e-09
		NSS	14.37	6.18	59.34	6.66e-15
		SS × NSS	0.29	0.17	4.05	2.21e-02
	S	SS	2.94	2.31	20.31	3.29e-06
		NSS	13.88	6.33	24.93	2.97e-07
		SS × NSS	0.39	0.17	13.55	1.16e-04
SI	D	SS	0.75	0.55	59.23	7.05e-15
		NSS	2.95	1.15	113.60	2.20e-16
		SS × NSS	0.06	0.02	25.32	2.43e-07
	S	SS	0.80	0.59	49.05	1.25e-12
		NSS	2.69	1.10	69.85	2.20e-16
		SS × NSS	0.05	0.02	36.23	8.76e-10
AN	D	SS	0.75	0.54	66.01	2.22e-16
		NSS	3.28	1.27	118.86	2.20e-16
		SS × NSS	0.08	0.02	37.19	5.35e-10
	S	SS	0.80	0.59	48.91	1.34e-12
		NSS	3.05	1.23	70.53	2.20e-16
		SS × NSS	0.06	0.02	43.59	2.03e-11

SS: GCA of Stiff Stalk (SS) inbred lines used as seed parents, reflecting additive effects; NSS: GCA of Non-Stiff Stalk (NSS) inbred lines used as pollen parents, reflecting additive effects; and SS × NSS: SCA of single-cross hybrids between SS and NSS inbred lines, reflecting dominance effects.

[1]This column indicates whether variance components were estimated from models specified with the **D** matrix (Class D) or with the **S** matrix (Class S).

[2]Presented p-values are given in scientific notation. Variance components showed different levels of significance, including $p < 0.05$, $p < 0.01$, and $p < 0.001$.

D: dominance genomic relationship matrix for single-cross hybrids derived from all possible crosses between parental inbred lines; **S**: covariance matrix derived from additive genetic relationships between parental inbred lines.

Estimates of variance components were obtained from the best-fitting GBLUP-based multi-kernel model (Equation 9).

Supplementary Table S11: Predictive ability and standard error for grain yield (GY, t ha^{-1}) across training set configurations and prediction methods.

Training Set Configuration	Method	Predictive Ability	Standard Error
T2	1a	0.735	0.031
T2	1b	0.760	0.024
T2	2a	0.756	0.029
T2	2b	0.780	0.021
T2	3a	0.757	0.030
T2	3b	0.776	0.022
T2	4a	0.768	0.022
T2	4b	0.787	0.019
T1F	1a	0.583	0.021
T1F	1b	0.562	0.018
T1F	2a	0.641	0.020
T1F	2b	0.618	0.018
T1F	3a	0.608	0.020
T1F	3b	0.637	0.017
T1F	4a	0.614	0.020
T1F	4b	0.636	0.017
T1M	1a	0.206	0.030
T1M	1b	0.248	0.030
T1M	2a	0.244	0.030
T1M	2b	0.245	0.031
T1M	3a	0.371	0.051
T1M	3b	0.411	0.036
T1M	4a	0.421	0.043
T1M	4b	0.447	0.034
T0	1a	-0.241	0.020
T0	1b	-0.273	0.018
T0	2a	-0.194	0.021
T0	2b	-0.272	0.018
T0	3a	0.510	0.015
T0	3b	0.514	0.013
T0	4a	0.512	0.013
T0	4b	0.512	0.013

T2 (both parents of the validation hybrid represented via other crosses), T1F (all hybrids sharing the same seed parent excluded), T1M (all hybrids sharing the same pollen parent excluded), and T0 (no direct parental information in the training set). Methods 1–2 correspond to GBLUP-based multi-kernel models (Equation 9), where the first includes only general combining ability (GCA) (Equation 11) and the second includes both GCA and specific combining ability (SCA) (Equation 12). Methods 3–4 are based on the covariance between tested and untested single-cross hybrids (Equation 13), considering either the additive relationship matrix only or both additive and non-additive relationship matrices. Results are shown for models fitted using either the **D** matrix (a) or the **S** matrix (b). **D**: dominance genomic relationship matrix for single-cross hybrids derived from all possible crosses between parental inbred lines; **S**: covariance matrix derived from additive genetic relationships between parental inbred lines. Additional model details are provided in Table 3.

Supplementary Table S12: Predictive ability and standard error for plant height (PH, cm) across training set configurations and prediction methods.

Training Set Configuration	Method	Predictive Ability	Standard Error
T2	1a	0.855	0.009
T2	1b	0.852	0.014
T2	2a	0.868	0.008
T2	2b	0.864	0.013
T2	3a	0.738	0.137
T2	3b	0.733	0.142
T2	4a	0.734	0.113
T2	4b	0.727	0.119
T1F	1a	0.767	0.010
T1F	1b	0.766	0.009
T1F	2a	0.789	0.010
T1F	2b	0.789	0.009
T1F	3a	0.415	0.019
T1F	3b	0.416	0.014
T1F	4a	0.401	0.018
T1F	4b	0.406	0.014
T1M	1a	0.159	0.025
T1M	1b	0.159	0.019
T1M	2a	0.170	0.025
T1M	2b	0.162	0.021
T1M	3a	0.241	0.013
T1M	3b	0.239	0.018
T1M	4a	0.260	0.012
T1M	4b	0.258	0.017
T0	1a	-0.100	0.022
T0	1b	-0.108	0.015
T0	2a	-0.093	0.022
T0	2b	-0.106	0.015
T0	3a	0.289	0.009
T0	3b	0.293	0.007
T0	4a	0.288	0.008
T0	4b	0.291	0.007

T2 (both parents of the validation hybrid represented via other crosses), T1F (all hybrids sharing the same seed parent excluded), T1M (all hybrids sharing the same pollen parent excluded), and T0 (no direct parental information in the training set). Methods 1–2 correspond to GBLUP-based multi-kernel models (Equation 9), where the first includes only general combining ability (GCA) (Equation 11) and the second includes both GCA and specific combining ability (SCA) (Equation 12). Methods 3–4 are based on the covariance between tested and untested single-cross hybrids (Equation 13), considering either the additive relationship matrix only or both additive and non-additive relationship matrices. Results are shown for models fitted using either the **D** matrix (a) or the **S** matrix (b). **D**: dominance genomic relationship matrix for single-cross hybrids derived from all possible crosses between parental inbred lines; **S**: covariance matrix derived from additive genetic relationships between parental inbred lines. Additional model details are provided in Table 3.

Supplementary Table S13: Predictive ability and standard error for ear height (EH, cm) across training set configurations and prediction methods.

Training Set Configuration	Method	Predictive Ability	Standard Error
T2	1a	0.805	0.013
T2	1b	0.801	0.014
T2	2a	0.816	0.012
T2	2b	0.816	0.014
T2	3a	0.767	0.065
T2	3b	0.783	0.058
T2	4a	0.758	0.056
T2	4b	0.772	0.054
T1F	1a	0.702	0.013
T1F	1b	0.700	0.013
T1F	2a	0.713	0.013
T1F	2b	0.718	0.013
T1F	3a	0.296	0.012
T1F	3b	0.294	0.013
T1F	4a	0.287	0.011
T1F	4b	0.287	0.013
T1M	1a	0.189	0.021
T1M	1b	0.188	0.018
T1M	2a	0.204	0.020
T1M	2b	0.204	0.017
T1M	3a	0.257	0.013
T1M	3b	0.265	0.015
T1M	4a	0.267	0.013
T1M	4b	0.274	0.015
T0	1a	-0.141	0.023
T0	1b	-0.147	0.028
T0	2a	-0.130	0.023
T0	2b	-0.128	0.028
T0	3a	0.195	0.006
T0	3b	0.198	0.006
T0	4a	0.192	0.005
T0	4b	0.196	0.006

T2 (both parents of the validation hybrid represented via other crosses), T1F (all hybrids sharing the same seed parent excluded), T1M (all hybrids sharing the same pollen parent excluded), and T0 (no direct parental information in the training set). Methods 1–2 correspond to GBLUP-based multi-kernel models (Equation 9), where the first includes only general combining ability (GCA) (Equation 11) and the second includes both GCA and specific combining ability (SCA) (Equation 12). Methods 3–4 are based on the covariance between tested and untested single-cross hybrids (Equation 13), considering either the additive relationship matrix only or both additive and non-additive relationship matrices. Results are shown for models fitted using either the **D** matrix (a) or the **S** matrix (b). **D**: dominance genomic relationship matrix for single-cross hybrids derived from all possible crosses between parental inbred lines; **S**: covariance matrix derived from additive genetic relationships between parental inbred lines. Additional model details are provided in Table 3.

Supplementary Table S14: Predictive ability and standard error for silking (SI, days) across training set configurations and prediction methods.

Training Set Configuration	Method	Predictive Ability	Standard Error
T2	1a	0.959	0.005
T2	1b	0.961	0.006
T2	2a	0.960	0.005
T2	2b	0.962	0.006
T2	3a	0.912	0.075
T2	3b	0.928	0.069
T2	4a	0.911	0.072
T2	4b	0.926	0.066
T1F	1a	0.707	0.009
T1F	1b	0.707	0.011
T1F	2a	0.721	0.009
T1F	2b	0.719	0.011
T1F	3a	0.572	0.003
T1F	3b	0.572	0.004
T1F	4a	0.567	0.003
T1F	4b	0.568	0.004
T1M	1a	0.646	0.021
T1M	1b	0.650	0.016
T1M	2a	0.648	0.020
T1M	2b	0.648	0.015
T1M	3a	0.504	0.015
T1M	3b	0.507	0.015
T1M	4a	0.517	0.015
T1M	4b	0.517	0.015
T0	1a	-0.554	0.008
T0	1b	-0.554	0.008
T0	2a	-0.544	0.009
T0	2b	-0.550	0.009
T0	3a	0.538	0.004
T0	3b	0.536	0.004
T0	4a	0.538	0.004
T0	4b	0.536	0.004

T2 (both parents of the validation hybrid represented via other crosses), T1F (all hybrids sharing the same seed parent excluded), T1M (all hybrids sharing the same pollen parent excluded), and T0 (no direct parental information in the training set). Methods 1–2 correspond to GBLUP-based multi-kernel models (Equation 9), where the first includes only general combining ability (GCA) (Equation 11) and the second includes both GCA and specific combining ability (SCA) (Equation 12). Methods 3–4 are based on the covariance between tested and untested single-cross hybrids (Equation 13), considering either the additive relationship matrix only or both additive and non-additive relationship matrices. Results are shown for models fitted using either the **D** matrix (a) or the **S** matrix (b). **D**: dominance genomic relationship matrix for single-cross hybrids derived from all possible crosses between parental inbred lines; **S**: covariance matrix derived from additive genetic relationships between parental inbred lines. Additional model details are provided in Table 3.

Supplementary Table S15: Predictive ability and standard error for anthesis (AN, days) across training set configurations and prediction methods.

Training Set Configuration	Method	Predictive Ability	Standard Error
T2	1a	0.950	0.008
T2	1b	0.949	0.007
T2	2a	0.951	0.008
T2	2b	0.950	0.007
T2	3a	0.896	0.083
T2	3b	0.894	0.085
T2	4a	0.894	0.077
T2	4b	0.893	0.080
T1F	1a	0.726	0.007
T1F	1b	0.722	0.011
T1F	2a	0.735	0.007
T1F	2b	0.732	0.011
T1F	3a	0.536	0.004
T1F	3b	0.536	0.005
T1F	4a	0.528	0.004
T1F	4b	0.529	0.005
T1M	1a	0.569	0.024
T1M	1b	0.566	0.024
T1M	2a	0.571	0.023
T1M	2b	0.564	0.024
T1M	3a	0.450	0.020
T1M	3b	0.436	0.020
T1M	4a	0.465	0.019
T1M	4b	0.450	0.019
T0	1a	-0.482	0.011
T0	1b	-0.488	0.008
T0	2a	-0.473	0.012
T0	2b	-0.486	0.008
T0	3a	0.493	0.005
T0	3b	0.491	0.004
T0	4a	0.493	0.005
T0	4b	0.490	0.004

T2 (both parents of the validation hybrid represented via other crosses), T1F (all hybrids sharing the same seed parent excluded), T1M (all hybrids sharing the same pollen parent excluded), and T0 (no direct parental information in the training set). Methods 1–2 correspond to GBLUP-based multi-kernel models (Equation 9), where the first includes only general combining ability (GCA) (Equation 11) and the second includes both GCA and specific combining ability (SCA) (Equation 12). Methods 3–4 are based on the covariance between tested and untested single-cross hybrids (Equation 13), considering either the additive relationship matrix only or both additive and non-additive relationship matrices. Results are shown for models fitted using either the **D** matrix (a) or the **S** matrix (b). **D**: dominance genomic relationship matrix for single-cross hybrids derived from all possible crosses between parental inbred lines; **S**: covariance matrix derived from additive genetic relationships between parental inbred lines. Additional model details are provided in Table 3.

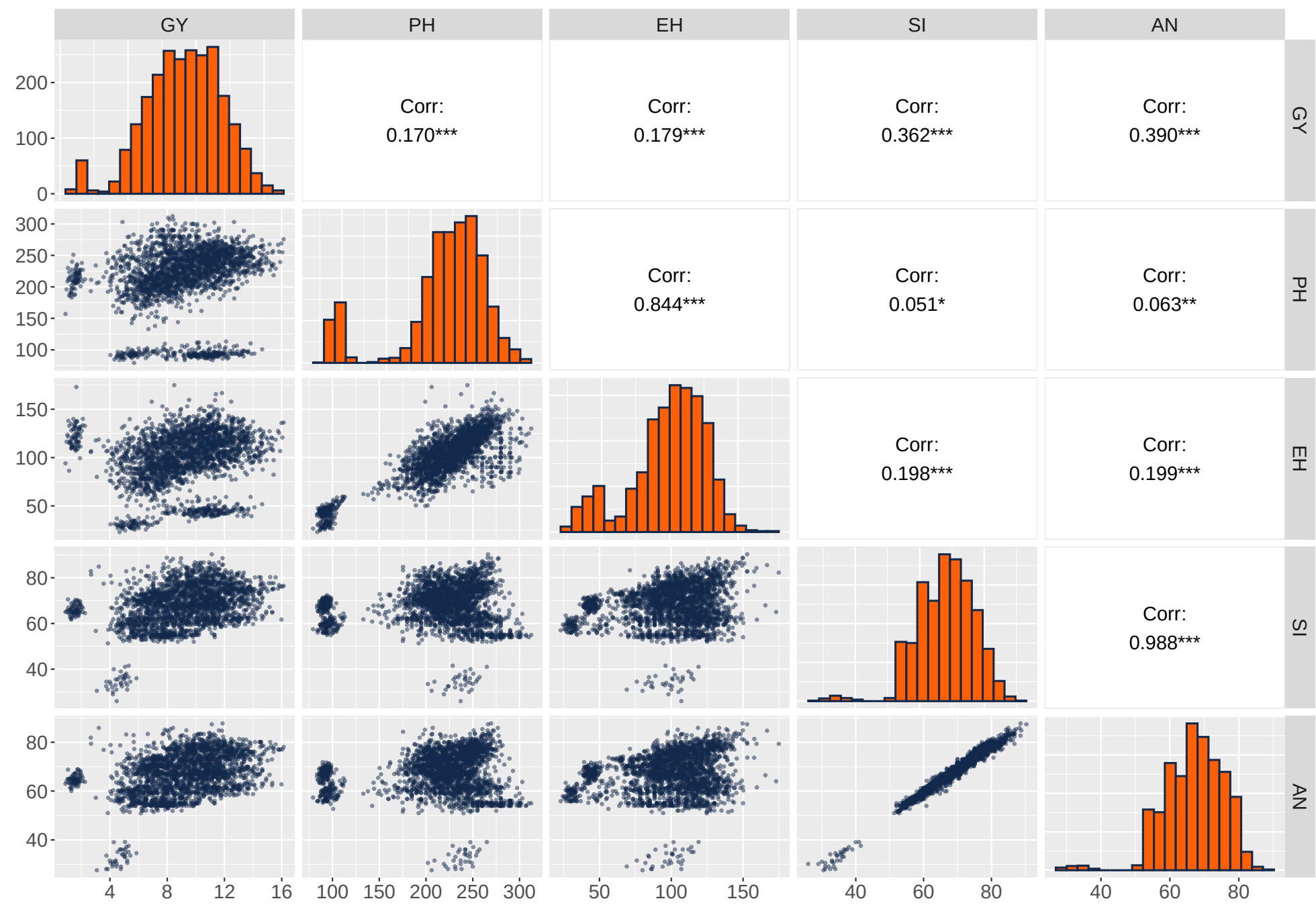

Supplementary Figure S1: Pairwise distribution and relationships among evaluated traits. Diagonal panels display histograms, lower panels show scatter plots, and upper panels present phenotypic correlations. Asterisks denote significance levels (*p-value < 0.05, **p-value < 0.01, and ***p-value < 0.001). Traits correspond to grain yield (GY, t ha^{-1}), plant height (PH, cm), ear height (EH, cm), silking (SI, days), and anthesis (AN, days).

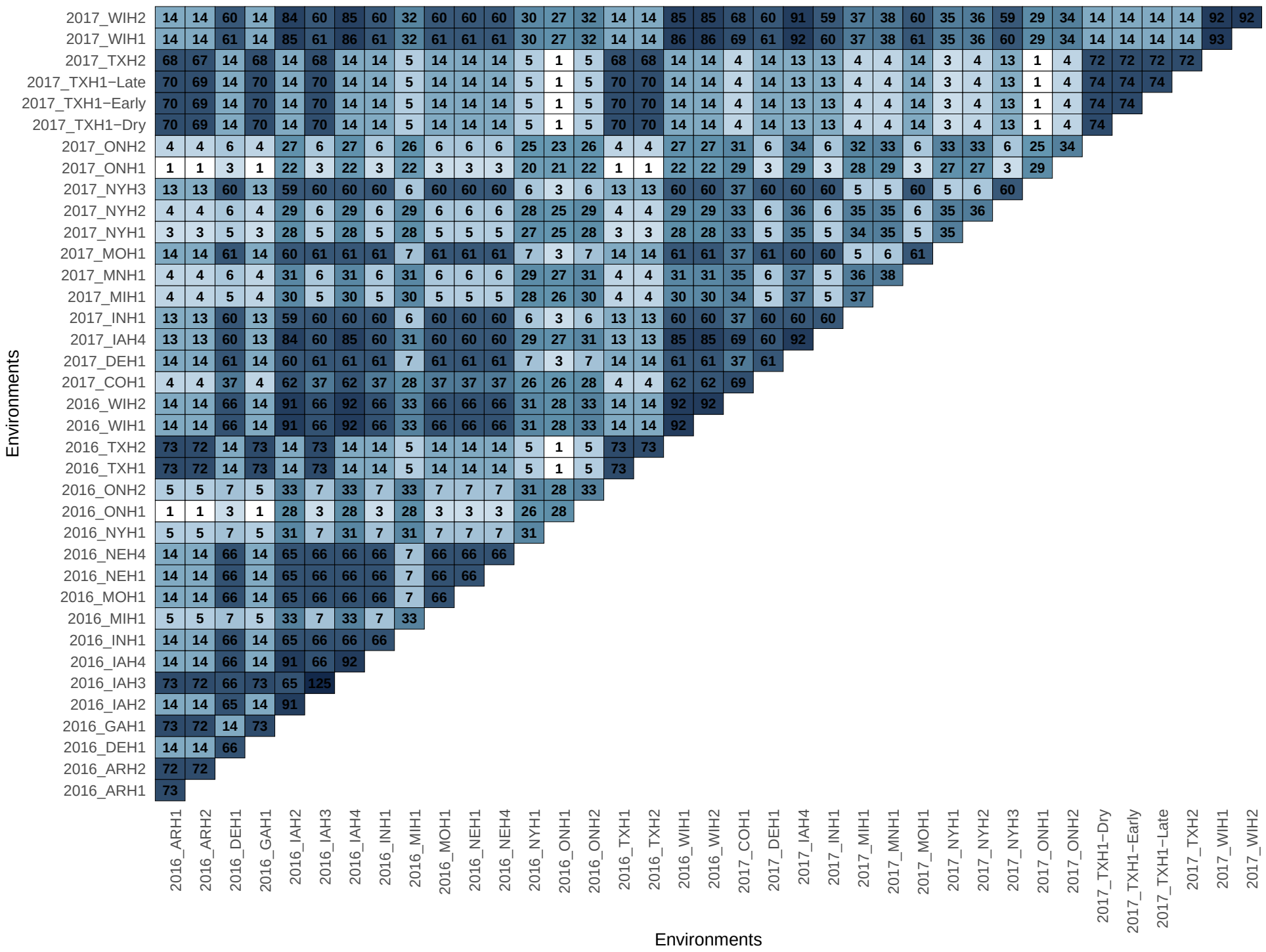

Supplementary Figure S2: Co-occurrence matrix of hybrids among environments. The diagonal values represent the number of unique hybrids in a given environment, whereas off-diagonal values represent the number of unique common hybrids among environments.

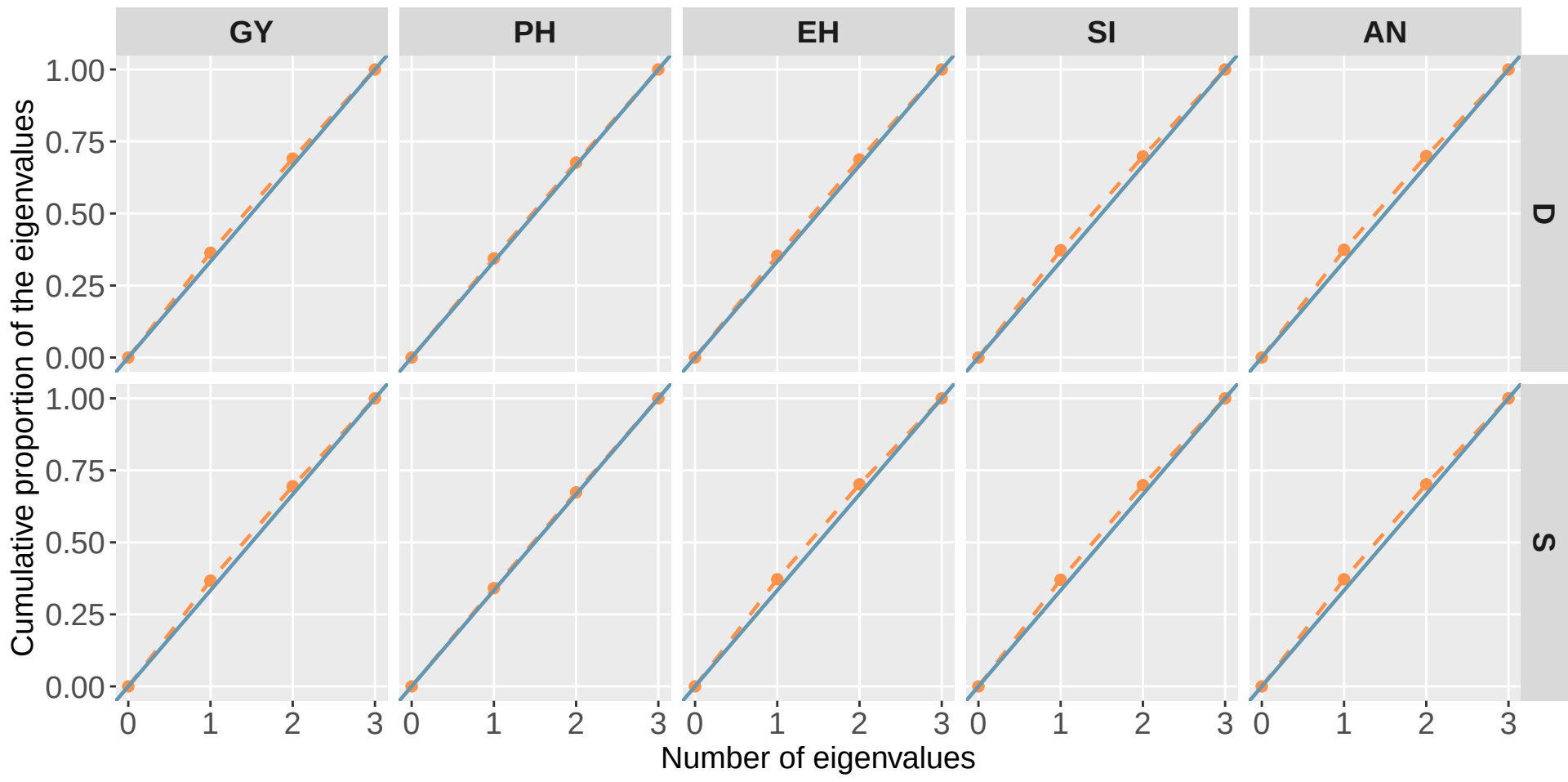

Supplementary Figure S3: Cumulative proportion (dashed line) of the relative contribution of the ordered eigenvalues derived from the asymptotic correlation matrices of variance components associated with the general combining ability (GCA) of Stiff Stalk (SS) and Non-Stiff Stalk (NSS) inbred lines, and the specific combining ability (SCA) of single-cross hybrids between SS and NSS inbred lines, for the early maturity group. The x-axis represents the rank of the eigenvalues, ordered from largest to smallest. The solid diagonal line represents the expected pattern under complete orthogonality (i.e., independence) among components, where all eigenvalues contribute equally and the cumulative proportion increases linearly. Deviations from this line indicate dependence among components. Results are shown for models fitted with either the **D** or **S** matrices in grain yield (GY, t ha^{-1}), plant height (PH, cm), ear height (EH, cm), silking (SI, days), and anthesis (AN, days). **D**: dominance genomic relationship matrix for single-cross hybrids derived from all possible crosses between parental inbred lines; **S**: covariance matrix derived from additive genetic relationships between parental inbred lines. The variance components were estimated from the GBLUP-based multi-kernel model described in Equation 9.

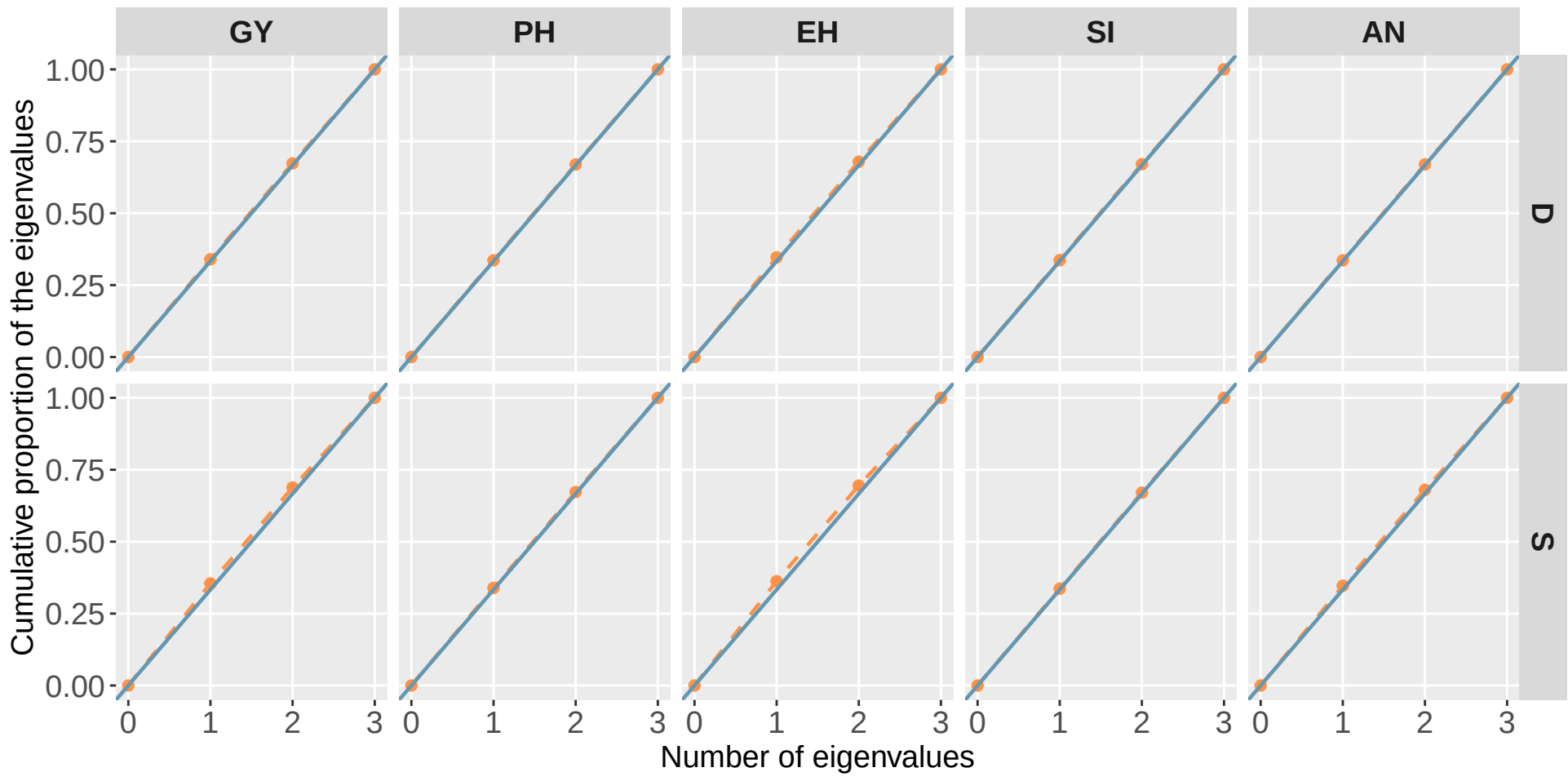

Supplementary Figure S4: Cumulative proportion (dashed line) of the relative contribution of the ordered eigenvalues derived from the asymptotic correlation matrices of variance components associated with the general combining ability (GCA) of Stiff Stalk (SS) and Non-Stiff Stalk (NSS) inbred lines, and the specific combining ability (SCA) of single-cross hybrids between SS and NSS inbred lines, for the late maturity group. The x-axis represents the rank of the eigenvalues, ordered from largest to smallest. The solid diagonal line represents the expected pattern under complete orthogonality (i.e., independence) among components, where all eigenvalues contribute equally and the cumulative proportion increases linearly. Deviations from this line indicate dependence among components. Results are shown for models fitted with either the **D** or **S** matrices in grain yield (GY, t ha^{-1}), plant height (PH, cm), ear height (EH, cm), silking (SI, days), and anthesis (AN, days). **D**: dominance genomic relationship matrix for single-cross hybrids derived from all possible crosses between parental inbred lines; **S**: covariance matrix derived from additive genetic relationships between parental inbred lines. The variance components were estimated from the GBLUP-based multi-kernel model described in Equation 9.

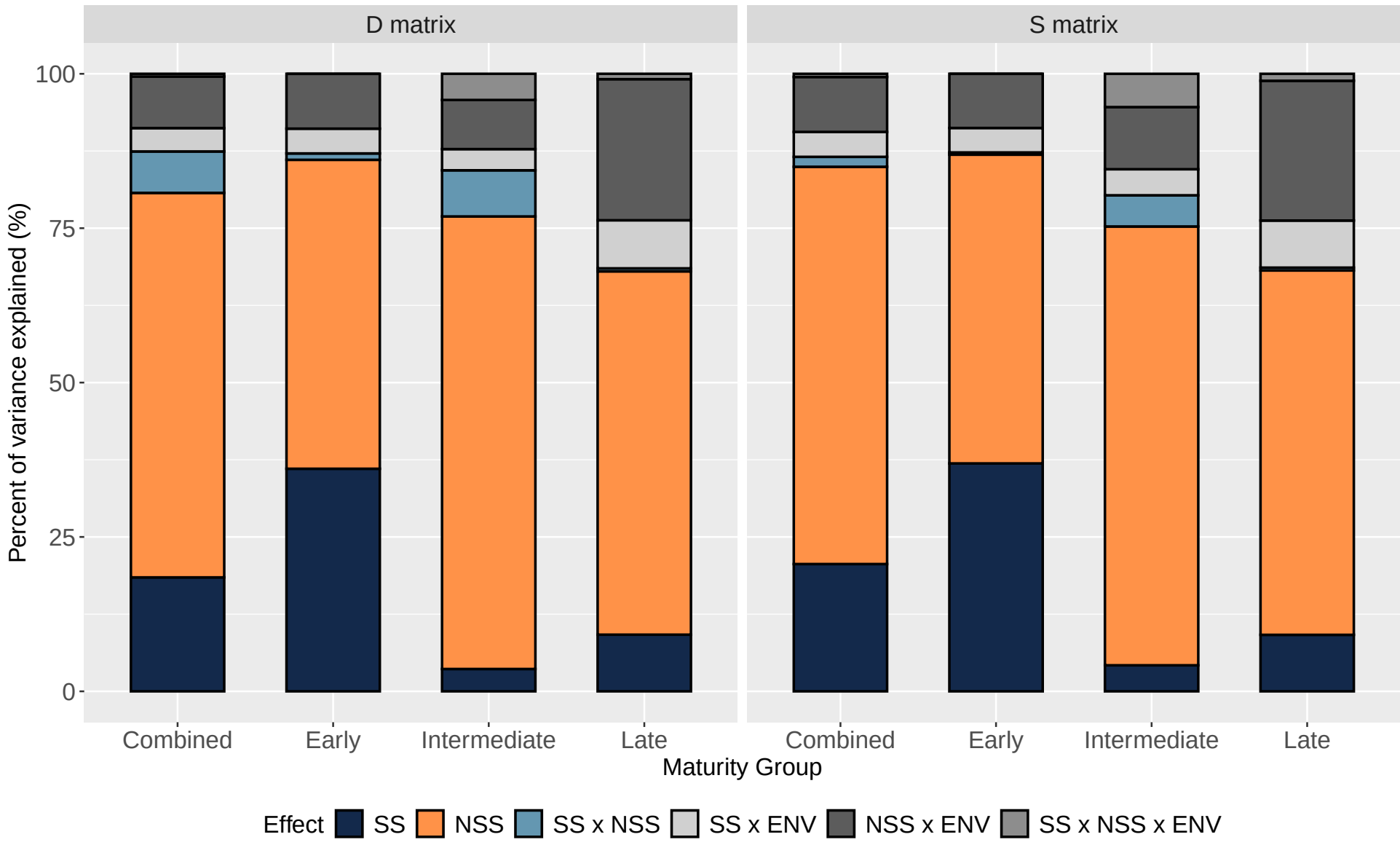

Supplementary Figure S5: Percent of variance explained for plant height (PH, cm). Variance components were estimated from the GBLUP-based multi-kernel model described in Equation 9, using either the **D** or the **S** matrices. SS: general combining ability (GCA) of Stiff Stalk (SS) inbred lines used as seed parents, reflecting additive effects; NSS: GCA of Non-Stiff Stalk (NSS) inbred lines used as pollen parents, reflecting additive effects; SS × NSS: specific combining ability (SCA) of single-cross hybrids between SS and NSS inbred lines, reflecting non-additive effects; SS × ENV: interaction between SS inbred lines and environments; NSS × ENV: interaction between NSS inbred lines and environments; SS × NSS × ENV: three-way interaction among SS, NSS, and environments, representing the interaction between SCA and environments; **D**: dominance genomic relationship matrix for single-cross hybrids derived from all possible crosses between parental inbred lines; **S**: covariance matrix derived from additive genetic relationships between parental inbred lines. Results are shown for all hybrids (Combined) as well as separately by maturity group (Early, Intermediate, Late).

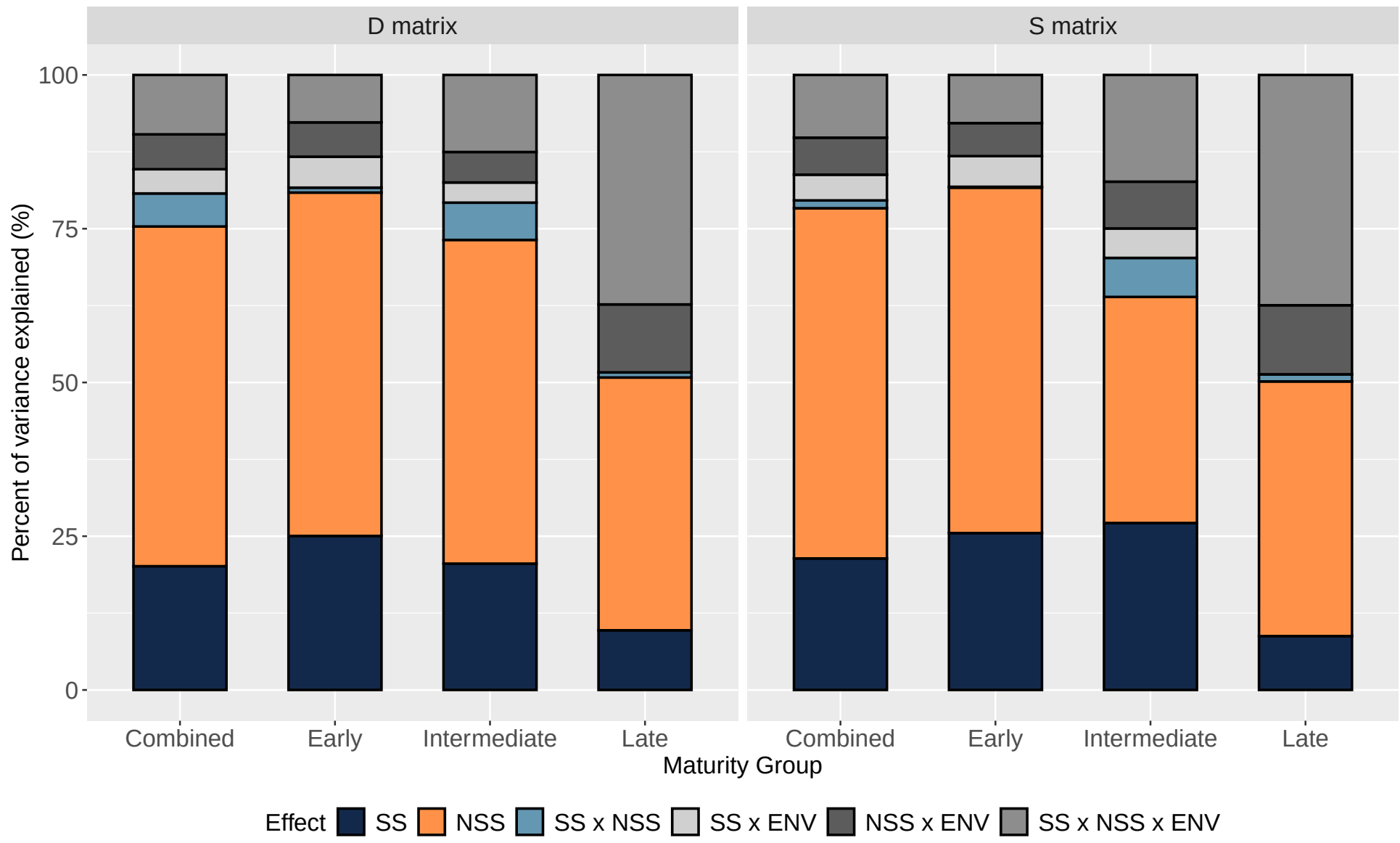

Supplementary Figure S6: Percent of variance explained for ear height (EH, cm). Variance components were estimated from the GBLUP-based multi-kernel model described in Equation 9, using either the **D** or the **S** matrices. SS: general combining ability (GCA) of Stiff Stalk (SS) inbred lines used as seed parents, reflecting additive effects; NSS: GCA of Non-Stiff Stalk (NSS) inbred lines used as pollen parents, reflecting additive effects; SS × NSS: specific combining ability (SCA) of single-cross hybrids between SS and NSS inbred lines, reflecting non-additive effects; SS × ENV: interaction between SS inbred lines and environments; NSS × ENV: interaction between NSS inbred lines and environments; SS × NSS × ENV: three-way interaction among SS, NSS, and environments, representing the interaction between SCA and environments; **D**: dominance genomic relationship matrix for single-cross hybrids derived from all possible crosses between parental inbred lines; **S**: covariance matrix derived from additive genetic relationships between parental inbred lines. Results are shown for all hybrids (Combined) as well as separately by maturity group (Early, Intermediate, Late).

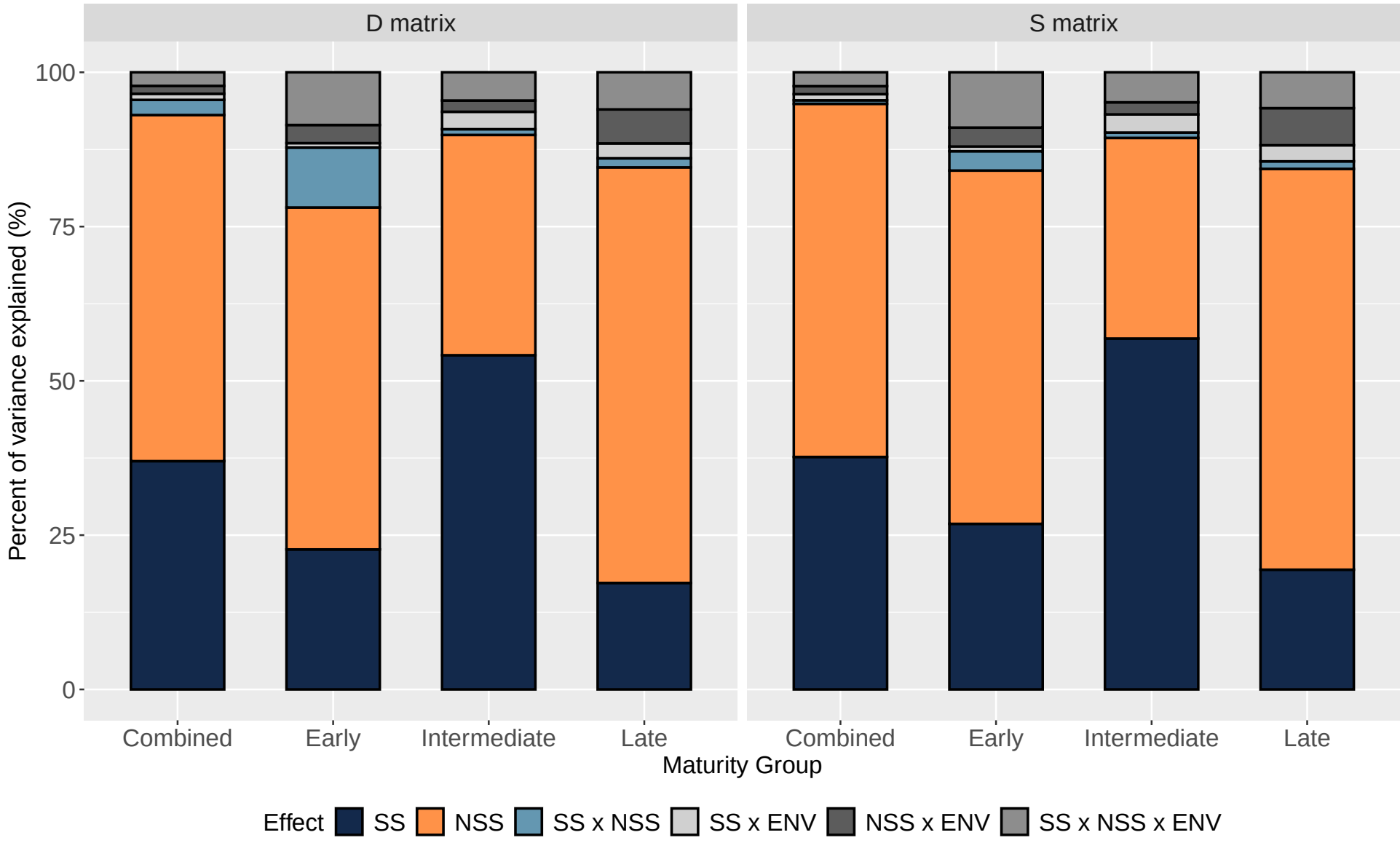

Supplementary Figure S7: Percent of variance explained for silking (SI, days). Variance components were estimated from the GBLUP-based multi-kernel model described in Equation 9, using either the **D** or the **S** matrices. SS: general combining ability (GCA) of Stiff Stalk (SS) inbred lines used as seed parents, reflecting additive effects; NSS: GCA of Non-Stiff Stalk (NSS) inbred lines used as pollen parents, reflecting additive effects; SS × NSS: specific combining ability (SCA) of single-cross hybrids between SS and NSS inbred lines, reflecting non-additive effects; SS × ENV: interaction between SS inbred lines and environments; NSS × ENV: interaction between NSS inbred lines and environments; SS × NSS × ENV: three-way interaction among SS, NSS, and environments, representing the interaction between SCA and environments; **D**: dominance genomic relationship matrix for single-cross hybrids derived from all possible crosses between parental inbred lines; **S**: covariance matrix derived from additive genetic relationships between parental inbred lines. Results are shown for all hybrids (Combined) as well as separately by maturity group (Early, Intermediate, Late).

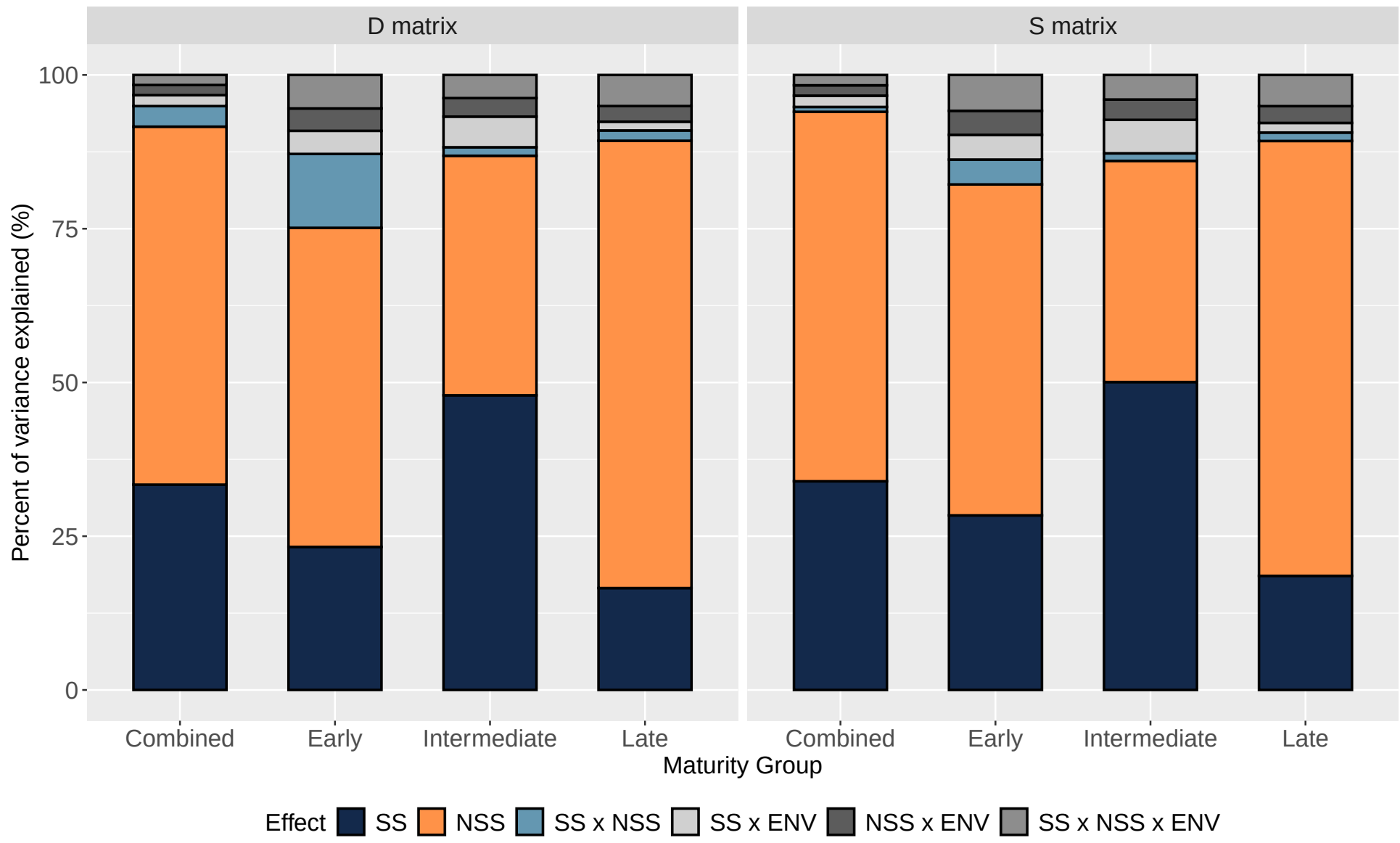

Supplementary Figure S8: Percent of variance explained for anthesis (AN, days). Variance components were estimated from the GBLUP-based multi-kernel model described in Equation 9, using either the **D** or the **S** matrices. SS: general combining ability (GCA) of Stiff Stalk (SS) inbred lines used as seed parents, reflecting additive effects; NSS: GCA of Non-Stiff Stalk (NSS) inbred lines used as pollen parents, reflecting additive effects; SS $\times$ NSS: specific combining ability (SCA) of single-cross hybrids between SS and NSS inbred lines, reflecting non-additive effects; SS $\times$ ENV: interaction between SS inbred lines and environments; NSS $\times$ ENV: interaction between NSS inbred lines and environments; SS $\times$ NSS $\times$ ENV: three-way interaction among SS, NSS, and environments, representing the interaction between SCA and environments; **D**: dominance genomic relationship matrix for single-cross hybrids derived from all possible crosses between parental inbred lines; **S**: covariance matrix derived from additive genetic relationships between parental inbred lines. Results are shown for all hybrids (Combined) as well as separately by maturity group (Early, Intermediate, Late).
